# Post-marketing safety of tarlatamab in small cell lung cancer based on FAERS and WHO-VigiAccess with SHAP-based interpretable machine learning analysis of immune-related adverse events

**DOI:** 10.3389/fphar.2026.1844248

**Published:** 2026-06-10

**Authors:** Yingyong Ou, Xianxian Zhong, Mei Mei, Xiao Gu, Shanshan Luo, Gaoyu Chen, Xuqin Xie, Desheng Sun

**Affiliations:** Department of Respiratory and Critical Care Medicine, Affiliated Hospital of Zunyi Medical University, Zunyi, Guizhou, China

**Keywords:** cytokine release syndrome, FAERS, immune effector cell-associated neurotoxicity syndrome, pharmacovigilance, shap, tarlatamab

## Abstract

**Background:**

Tarlatamab is a DLL3-targeted bispecific T-cell engager approved for previously treated extensive-stage small cell lung cancer (ES-SCLC). However, its post-marketing safety profile in routine practice remains incompletely characterized.

**Methods:**

We conducted a retrospective pharmacovigilance study using the FDA Adverse Event Reporting System from Q2 2024 to Q3 2025, with a descriptive cross-database comparison using WHO-VigiAccess. Disproportionality analyses were performed using reporting odds ratio, proportional reporting ratio, Bayesian confidence propagation neural network, and multi-item gamma Poisson shrinker algorithms. Clinical priority scoring, subgroup analysis, time-to-onset analysis, multivariable logistic regression, and interpretable machine learning with SHAP (SHapley Additive exPlanations) were further applied.

**Results:**

A total of 942 reports with tarlatamab as the primary suspect drug were identified, comprising 1,346 adverse events. At the preferred-term level, 30 signals met all four disproportionality criteria. The most frequent and strongest signals were cytokine release syndrome (CRS; n = 201; ROR 223.84, 95% CI 192.15–260.77) and immune effector cell-associated neurotoxicity syndrome (ICANS; n = 106; ROR 312.43, 95% CI 254.68–383.26). Common additional signals included pyrexia, dysgeusia, hypotension, and ageusia. Potentially under-recognized events, such as intestinal perforation, dyspnoea at rest, and incontinence, were also detected. Most adverse events occurred early after treatment initiation, with a median time to onset of 3 days; CRS and ICANS both showed a median onset of 1 day. In multivariable analysis, concomitant medication use was associated with higher reported odds of CRS (OR 2.551, 95% CI 1.353–4.811), whereas reports from Japan and the year 2025 were associated with lower reported odds. The CRS report-level classification model showed acceptable discrimination (AUC 0.733), whereas the best machine-learning model for adverse events of immune disorders classification within FAERS reports showed only modest performance (validation AUC 0.639). SHAP analysis indicated that country and therapy type contributed more to model output than age and sex, suggesting that the model primarily captured reporting context and treatment complexity rather than intrinsic biological susceptibility. Cross-database comparison with WHO-VigiAccess showed a broadly concordant reporting pattern, with CRS and ICANS remaining the most commonly reported toxicities.

**Conclusion:**

Post-marketing data indicate that tarlatamab has a distinct safety profile dominated by early-onset immune-mediated and neurologic toxicities, particularly CRS and ICANS. Early monitoring and prompt supportive management during initial treatment cycles are essential. These findings broaden current knowledge of tarlatamab safety in real-world practice and support prospective studies to pharmacovigilance-based signal stratification and monitoring strategies.

## 1 Introduction

Small cell lung cancer (SCLC) is a highly aggressive neuroendocrine malignancy characterized by rapid proliferation, early metastatic spread, and poor prognosis. It accounts for approximately 14% of all lung cancers, and 60%–70% of patients present with extensive-stage disease at initial diagnosis. The incidence of brain metastases during the disease course reaches up to 40%–50%, and the median overall survival for extensive-stage SCLC remains limited to 6–12 months (Bolte et al., 2025). Despite advances in surgery, radiotherapy, chemotherapy, and immunotherapy (Kemper et al., 2025), durable disease control remains uncommon, highlighting a critical unmet need for novel targeted therapies.

Tarlatamab (tarlatamab-dlle; IMDELLTRA™) is a novel half-life–extended bispecific T-cell engager (BiTE) antibody that simultaneously targets Delta-like ligand 3 (DLL3) on tumor cells and CD3 on cytotoxic T lymphocytes (CTLs). By physically bridging tumor cells and T cells, tarlatamab induces T-cell activation, promotes cytokine release, and facilitates targeted cytotoxicity against DLL3-expressing malignancies (Dhillon, 2024). Under physiological conditions, DLL3 is an intracellular protein involved in the negative regulation of the Notch signaling pathway; however, it is aberrantly expressed on the surface of SCLC cells. Notably, DLL3 expression has been reported in approximately 85%–94% of patients with SCLC, making it an attractive therapeutic target (Ahn et al., 2023).

In May 2024, tarlatamab received its first approval from the U.S. Food and Drug Administration (FDA) for adult patients with extensive-stage SCLC (ES-SCLC) who have progressed on or after platinum-based chemotherapy (Dhillon, 2024). Emerging evidence suggests that tarlatamab may also have therapeutic potential in other neuroendocrine malignancies, including neuroendocrine prostate cancer and head and neck neuroendocrine carcinoma (Aggarwal et al., 2025). Given its unique mechanism of action and promising efficacy, tarlatamab represents an important advancement in the treatment landscape of SCLC. However, the immune activation induced by BiTE therapies is inherently associated with immune-related toxicities. Clinical trials, including DeLLphi-301 and DeLLphi-303, have reported a high incidence of adverse events, particularly cytokine release syndrome (CRS), pyrexia, decreased appetite, dysgeusia, anemia, and immune effector cell-associated neurotoxicity syndrome (ICANS) (Sands et al., 2025; Lawrence, 2026). Current evidence regarding the safety profile of tarlatamab is largely derived from clinical trials, meta-analyses, and case reports. These studies are limited by strict inclusion criteria, relatively small sample sizes, and short follow-up durations, which may not fully reflect real-world safety outcomes. In particular, immune-related adverse events may lead to prolonged hospitalization, increased susceptibility to infection, or even death (Bajwa et al., 2025). Therefore, comprehensive post-marketing pharmacovigilance studies based on real-world data are essential to better characterize its safety profile.

Traditional pharmacovigilance approaches, such as disproportionality analysis, are effective for detecting population-level safety signals but provide limited insight into individual-level risk prediction. Recently, interpretable machine learning (ML) has emerged as a promising complementary strategy, enabling improved predictive performance while maintaining model transparency. This approach has been successfully applied in multiple biomedical domains, including drug discovery, proteomics, and genomics (Medina-Ortiz et al., 2025). These advances may offer new opportunities for exploring report-level patterns related to tarlatamab-associated immune adverse events and improving the interpretability of pharmacovigilance-based signal stratification (Liu et al., 2024). In this study, we conducted a comprehensive pharmacovigilance analysis based on the U.S. Food and Drug Administration (FDA) Adverse Event Reporting System (FAERS) database, complemented by descriptive cross-database comparison using the WHO VigiAccess platform. By integrating disproportionality analysis with interpretable ML, we aimed to systematically evaluate the safety profile of tarlatamab and provide evidence to support its rational clinical use.

## 2 Methods

### 2.1 Data collection, FAERS database description, and MedDRA terminology

**FIGURE 1:**
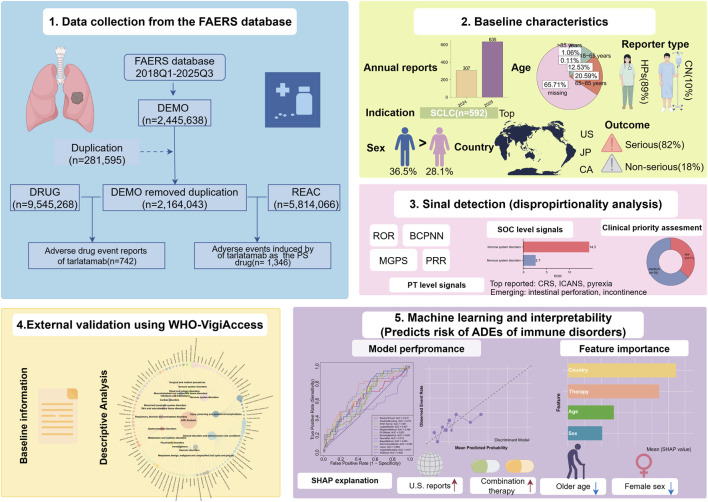
Flowchart of the entire study.

This study conducted a retrospective pharmacovigilance analysis based on the FAERS database. Post-marketing adverse event reports submitted between the second quarter of 2024 and the third quarter of 2025 were included. Given that tarlatamab received its first FDA approval in May 2024, this study period captures both early post-marketing and recent real-world safety data. The FAERS database comprises multiple data files, including DEMO, DRUG, REAC, OUTC, RPSR, THER, and INDI, which respectively contain patient demographic information, drug exposure data, adverse event reports, clinical outcomes, report sources, therapy dates, and indications. In this study, the PRIMARY ID was used as the unique identifier to link and integrate records across these datasets. Reports associated with the target drug were identified by searching the DRUG file using both generic names (“TARLATAMAB”, “TARLATAMAB DLLE”) and the brand name (“IMDELLTRA”). As the FAERS database relies on spontaneous reporting, duplicate or withdrawn/deleted reports are inevitable. 1n this study, reports were excluded if tarlatamab was not explicitly identified as the primary suspected (role_code: PS) drug, if adverse event descriptions were vague or critical information was missing (e.g., lacking specific event names or showing no temporal relationship with drug administration), or if cases shared identical events, event dates, age, sex, and country of origin (duplicate report) (Cirmi et al., 2020). Following FDA guidance on data refinement, duplicates were systematically removed using a hierarchical protocol: unique identifiers (PRIMARY ID), case identifiers (CASEID), and FDA receipt dates (FDA_DT) were extracted from the DEMO file and sorted by CASEID, FDA_DT, and PRIMARY ID; for duplicate CASEIDs, the report with the most recent FDA_DT was retained, and when CASEID and FDA_DT were identical, the entry with the highest PRIMARY ID was selected. An additional refinement step was applied by cross-referencing reports with the quarterly “deleted files” list, implemented since the first quarter of 2019, to remove withdrawn reports and further enhance dataset accuracy (Sui et al., 2025). In our analysis, missing categorical variables were not imputed. To avoid selection bias resulting from excluding cases with incomplete information, all 942 unique case reports were retained in the analysis cohort. During the generation of descriptive statistics, missing values were automatically treated as a separate “missing” category. This approach ensures transparency in data completeness and aligns with standard practices in pharmacovigilance research (Shen et al., 2025). Adverse events were coded using the Medical Dictionary for Regulatory Activities (MedDRA), version 28.1 (Brown, 2003). Preferred Terms (PTs) and their corresponding System Organ Classes (SOCs) were standardized and mapped accordingly. After data preprocessing, a total of 2,164,043 DEMO records, 9,545,268 DRUG records, and 5,814,066 REAC records were obtained. Among these, 942 reports identified tarlatamab as the primary suspect drug, encompassing 1,346 tarlatamab-related adverse events. The study workflow is illustrated in Figure 1.

### 2.2 Disproportionality analysis

Disproportionality analysis is a classical approach in pharmacovigilance studies for identifying potential associations between drugs and specific adverse drug events (ADEs) (Scosyrev et al., 2025). The fundamental principle involves comparing the reporting proportion of a specific ADE associated with the target drug to that observed in a reference group, in order to determine whether the ADE is reported more frequently than expected among individuals exposed to the target drug. A significantly higher reporting proportion suggests the presence of a potential disproportionality signal.

In this study, four widely used pharmacovigilance signal detection methods were employed, including the reporting odds ratio (ROR), proportional reporting ratio (PRR), Bayesian confidence propagation neural network (BCPNN), and multi-item gamma Poisson shrinker (MGPS) algorithms. ROR and PRR are traditional ratio-based methods, characterized by computational simplicity, intuitive interpretation, and strong transparency (van Puijenbroek et al., 2002; Trippe et al., 2017). BCPNN reduces the impact of data sparsity on estimate stability and is particularly suitable for detecting rare adverse event signals (Bate, 2007). MGPS, through Bayesian shrinkage, enhances control of the signal-to-noise ratio, thereby improving both the accuracy and sensitivity of signal detection (Ahlmann-Eltze and Huber, 2021). Given that each algorithm has distinct strengths in signal identification, all four methods were applied in combination to improve the comprehensiveness and robustness of the analysis (Chen et al., 2025). All signal calculations were based on 2 × 2 contingency tables (Table 1). The criteria for defining a positive signal were as follows: for ROR, the lower limit of the 95% confidence interval (CI) > 1 with a minimum of three co-occurrences (n ≥ 3); for PRR, PRR >2 with χ^2^ ≥ 4; for BCPNN, the 2.5th percentile of the information component (IC025) > 0; and for MGPS, the lower limit of the 95% CI of the empirical Bayesian geometric mean (EBGM05) > 2. These thresholds were established according to commonly accepted pharmacovigilance practices. In general, higher values of these metrics indicate a stronger potential association between the target drug and the corresponding adverse event, reflecting greater signal strength.

**TABLE 1:** We summarize the methodologies, corresponding formulas, and signal detection thresholds applied for the reporting odds ratio (ROR), proportional reporting ratio (PRR), Bayesian confidence propagation neural network (BCPNN), and empirical Bayesian geometric mean (EBGM).

​	Target adverse drug event	Non-target adverse drug event	Sums
Tarlatamab	a	b	a + b
Non-tarlatamab	c	d	c + d
Total	a + c	b + d	a + b + c + d

Methods	Formula	Threshold
ROR	$ROR=\frac{a/c}{b/d}$	a≥3
	$SE\left(lnROR\right)=\sqrt{\left(\frac{1}{a}+\frac{1}{b}+\frac{1}{c}+\frac{1}{d}\right)}$	​
	$95\%\text{CI}=e^{\ln\left(\text{ROR}\right)\pm 1.96\text{se}}$	$95\%\text{CI}\left(\text{lower limit}\right)>1$
PRR	$\text{PRR}=\frac{a/\left(a+b\right)}{c/\left(c+d\right)}$	a≥3
	χ^2^ = [(ad - bc)^2^](a + b + c + d)/[(a + b) (c + d) (a + c) (b + d)]	PRR>2 χ2≥4
BCPNN	$\text{IC}=\log_{2}\frac{p\left(x,y\right)}{p\left(x\right)p\left(y\right)}=\log_{2}\frac{a\left(a+b+c+d\right)}{\left(a+b\right)\left(a+c\right)}$	IC025>0
	$E\left(\text{IC}\right)=\log_{2}\frac{\left(a+\gamma 11\right)\left(a+b+c+d+\alpha \right)\left(a+b+c+d+\beta \right)}{\left(a+b+c+d+\gamma \right)\left(a+b+\alpha 1\right)\left(a+c+\beta 1\right)}$	
	$V\left(\text{IC}\right)=\frac{1}{{\left(\ln2\right)}^{2}}\left[\frac{\left(a+b+c+d\right)-a+\gamma -\gamma 11}{\left(a+\gamma 11\right)\left(1+a+b+c+d+\gamma \right)}+\frac{\left(a+b+c+d\right)-\left(a+b\right)+a-\alpha 1}{\left(a+b+\alpha 1\right)\left(1+a+b+c+d+\alpha \right)}+\frac{\left(a+b+c+d+\alpha \right)-\left(a+c\right)+\beta -\beta 1}{\left(a+b+\beta 1\right)\left(1+a+b+c+d+\beta \right)}\right]$	
	$\gamma =\gamma 11\frac{\left(a+b+c+d+\alpha \right)\left(a+b+c+d+\beta \right)}{\left(a+b+\alpha 1\right)\left(a+c+\beta 1\right)}$	
	$\text{IC}-2\text{SD}=E\left(\text{IC}\right)-2\sqrt{V\left(\text{IC}\right)}$	
EBGM	$\text{EBGM}=\frac{a\left(a+b+c+d\right)}{\left(a+c\right)\left(a+b\right)}$	EBGM05>2
	$\text{SE}\left(\text{lnEBGM}\right)=\sqrt{\left(\frac{1}{a}+\frac{1}{b}+\frac{1}{c}+\frac{1}{d}\right)}$	
	$95\%\text{CI}=e^{\ln\left(\text{EBGM}\right)\pm 1.96\text{se}}$	

In this 2 × 2 contingency table, a denotes the number of reports containing both tarlatamab and the target adverse event; b represents the number of reports involving tarlatamab in association with other adverse events; c refers to the number of reports describing the target adverse event in relation to other drugs; and d indicates the number of reports involving other drugs combined with non-target adverse events.

95% CI, 95% confidence interval; χ², chi-squared statistic; IC, information component; IC025, 2.5th percentile of the information component; E(IC), expected value of IC; V(IC), variance of IC; and EBGM05, 5th percentile of the empirical Bayes geometric mean.

### 2.3 Clinical priority assessment

In this study, a semi-quantitative evaluation framework was developed to prioritize clinically relevant ADEs, based on the guidance of the European Medicines Agency (EMA) regarding Designated Medical Events (DMEs) and Important Medical Events (IMEs) (Cecco et al., 2024). This framework integrates both the clinical significance of ADEs and the robustness of the detected signals, enabling a stratified assessment of different adverse events while also facilitating the identification of potential inconsistencies in reporting patterns. According to the composite scoring system, all ADEs were categorized into three priority levels: low priority (0–2 points), moderate priority (3–5 points), and high priority (6–10 points). Detailed scoring criteria and classification standards are presented in Table 2.

**TABLE 2:** Criteria and scoring framework applied to prioritize adverse drug events detected *via* disproportionality analysis.

Criterium	2 points	1 point	0 point
Reporting rate (cases/non-cases)	>10%	1–10%	0–1%
Signal stability (consistency across disproportionality analyses)	3/4 of 4	2 of 3	1 of 3
Reported case fatality rate (proportion of reports with death as outcome)	>50%	25–50%	<25%
Clinical relevance (serious likely drug-attributable ADEs)	DME	IME	None

ADEs, adverse drug events; DME designated medical event; IME important medical event.

### 2.4 Machine learning modeling and model interpretation

To explore whether report-level characteristics could help classify immune-related adverse event reports associated with tarlatamab, multiple MLs algorithms were applied for model construction and performance evaluation. The primary ML task is the binary classification of tarlatamab-associated “immune disorders” in SOCs, specifically distinguishing between reported (positive class) and unreported immune disorders (negative class) associated with tarlatamab. Prior to analysis, categorical variables (such as sex) were processed using one-hot encoding. For continuous features (such as age), Z-score standardization was performed to ensure that each feature had a mean of 0 and a standard deviation of 1, facilitating comparison across features with different units of measurement. This study utilized 13 commonly used supervised learning algorithms, including logistic regression, Lasso regression, discriminant analysis, support vector machine (SVM), random forest, gradient boosting, extreme gradient boosting (XGBoost), neural networks, Bayesian methods, and k-nearest neighbor algorithms. The dataset was randomly split into a training set and a validation set in a 1:1 ratio. To mitigate overfitting and improve generalizability, 5-fold cross-validation with 10 repetitions was employed on the training set, and grid search was used to optimize the hyperparameters of all models (Chen et al., 2024; Wu et al., 2025). The final models were trained using the best-performing hyperparameters, and evaluation was conducted on the validation set. The optimal hyperparameters after the 5-fold cross-validation for each ML model are provided in Supplementary Table S1. Model performance was rigorously quantified using metrics such as area under the receiver operating characteristic (ROC) curve (AUC), sensitivity, specificity, accuracy, and F1 score. The discriminatory ability of the models was assessed by the AUC and its 95% confidence interval (CI), with results visualized using forest plots. Additionally, confusion matrices were constructed to evaluate classification performance in both the training and validation sets, and overall accuracy was calculated. To further assess the consistency between predicted probabilities and observed outcomes, calibration curves were generated to evaluate model calibration performance. The selection of the best model was based on its overall performance on the independent validation set. A higher AUC under the ROC curve was the primary criterion for selecting the optimal model, with F1 score, sensitivity, and specificity serving as secondary metrics for evaluating model performance (Yang et al., 2026). After identifying the best-performing model, the SHAP (SHapley Additive exPlanations) method was integrated to enhance model interpretability (Li Y. et al., 2025). By calculating the SHAP values for each feature, we precisely quantified the contribution of each feature to the model’s predictions, providing a practical and interpretable example of ML-based feature identification. First, feature importance was assessed by calculating the mean absolute SHAP value for each variable, followed by ranking the features. SHAP beeswarm plots were then used to illustrate the direction and magnitude of each feature’s contribution to the model’s predictions across different value ranges. Additionally, dependence plots were generated to explore the relationships between key variables (e.g., age, sex, country, and therapy type) and predicted risk. At the individual level, waterfall plots and force plots were employed to visualize the contribution of each feature to the prediction for a single sample, thereby offering interpretable insights into the model’s decision-making process.

### 2.5 Logistic regression analysis

To investigate the risk of CRS associated with tarlatamab and to identify potential risk factors for mortality following CRS onset, univariate and multivariate logistic regression models were constructed. Independent variables included age, sex, reporter type, treatment regimen (with or without concomitant medications), reporting country, and reporting year. To ensure the reliability of the analysis, reports with missing values in any of these variables were excluded. For the purpose of statistical modeling, the original count data based on System Organ Class (SOC) were transformed into binary outcome variables. Specifically, for the analysis of CRS occurrence, “CRS following tarlatamab use” was coded as 1, and “no CRS occurrence” as 0. For the mortality risk analysis, “death following CRS” was coded as 1, whereas “survival after CRS” was coded as 0.

### 2.6 Time-to-onset (TTO) analysis

In this study, TTO was defined as the interval between the date of adverse event occurrence (recorded in the DEMO file) and the initiation date of drug therapy (recorded in the THER file). Reports with invalid date records (e.g., erroneous entries or incomplete date information lacking year, month, or day) or negative calculated intervals were excluded to ensure analytical accuracy. At the overall level, TTO was summarized using the median and interquartile range (IQR) to describe its central tendency and dispersion. In addition, the temporal distribution of TTO was characterized using the shape parameter (β) of the Weibull distribution model. In Weibull analysis, a β value <1 with a 95% confidence interval (CI) entirely below 1 indicates a decreasing hazard over time, corresponding to an “early failure” type; a β value approximately equal to 1 with a 95% CI including 1 suggests a constant hazard over time, consistent with a “random failure” type; and a β value >1 with a 95% CI excluding 1 indicates an increasing hazard over time, corresponding to a “wear-out failure” type (Shu et al., 2022). At the PT level, differences in TTO among various PTs were compared using the Kruskal–Wallis rank-sum test. Furthermore, Kaplan–Meier survival analysis was applied to estimate the cumulative incidence of adverse events across different subgroups, with comparisons performed between groups.

### 2.7 Cross-database descriptive comparison using the WHO VigiAccess

The data used in this study were obtained from VigiAccess (https://www.vigiaccess.org/), a publicly accessible online platform developed by the World Health Organization (WHO). This platform is based on VigiBase, the WHO global database of individual case safety reports (ICSRs), and provides aggregated information on ADEs related to medicines and vaccines. On 31 January 2026, we accessed the platform and systematically retrieved adverse event reports associated with tarlatamab. The extracted data included key demographic characteristics of the reporting population, such as age groups, sex distribution, reporting year, and geographic distribution by continent. All adverse events were coded using the Medical Dictionary for Regulatory Activities (MedDRA) and were categorized and analyzed according to SOCs and PTs.

### 2.8 Statistical analysis and software

All statistical analyses and data visualizations were performed using R software (version 4.2.3). All statistical tests were two-sided, with a *P* value <0.05 considered statistically significant. The study design and reporting were conducted in accordance with the READUS-PV guidelines, ensuring transparency and reproducibility of the research methodology and findings (Fusaroli et al., 2024).

## 3 Results

### 3.1 Description of baseline information from adverse reaction reports relating to tarlatamab in the FAERS database

**FIGURE 2:**
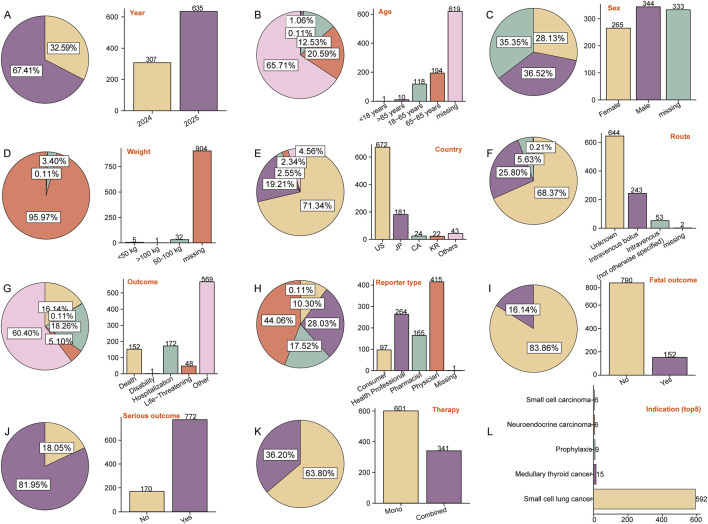
Clinical characteristics of ADE reports associated with tarlatamab. **(A)** Year of report submission. **(B)** Age of reporters. **(C)** Sex distribution. **(D)** Body weight. **(E)** Country of origin of the report. **(F)** Administration route. **(G)** Outcome of the adverse event. **(H)** Reporter type. **(I)** Fatal outcome of the event. **(J)** Serious outcome of the event. **(K)** Therapy type. **(L)** Medical indications for tarlatamab use. Kg, kilogram; US, United States; JP, Japan; CA, Canada; KR, Korean.

A detailed analysis of the baseline characteristics of 942 ADE reports associated with tarlatamab in the FAERS database was performed. As of the third quarter of 2025, the number of reports in 2025 (635 reports) had more than doubled compared to 2024 (307 reports) (Figure 2A). Among reports with specific age information, the majority of reporters were aged 65–85 years (n = 194, 20.59%), followed by the middle-aged group (18–65 years) (n = 118, 12.53%) (Figure 2B). In reports containing sex information, male reporters accounted for a higher proportion (n = 344, 36.52%) compared to female reporters (n = 265, 28.13%) (Figure 2C). However, over 90% of the reports lacked detailed weight information (n = 904, 95.97%) (Figure 2D). Regarding the reporting countries, the top four countries by report count were the United States (n = 672), Japan (n = 181), Canada (n = 24), and South Korea (n = 22) (Figure 2E). In terms of route of administration, intravenous injection was the most common (Figure 2F), consistent with the drug’s label. Among reports that included outcome information, the top three outcomes were hospitalization (n = 172, 18.26%), death (n = 152, 16.14%), and life-threatening (n = 48, 5.10%) (Figure 2G). Regarding the type of reporter, the majority of reports were submitted by physicians (n = 415, 44.06%) and healthcare professionals (n = 264, 28.09%), with reports from consumers accounting for 10.30% (Figure 2H). The proportions of death and severe outcomes were 16.14% (Figure 2I) and 81.95% (Figure 2J), respectively. Most reports did not include information on concomitant medications (n = 601, 63.80%) (Figure 2K). The most common recorded indication was SCLC (n = 592), which is consistent with the approved indication for tarlatamab (Figure 2L). Because missingness varied substantially across variables, different analytic subsets were used for different downstream analyses. Descriptive summaries were based on all available reports for each variable, whereas subgroup, regression, and machine-learning analyses were restricted to reports with complete data for the variables required by each analysis. Body weight was not used in subgrouping or predictive modeling because 95.97% of reports lacked weight information.

### 3.2 Signal value detection based on the disproportionate analysis

#### 3.2.1 Signal detection at the SOC level

**FIGURE 3:**
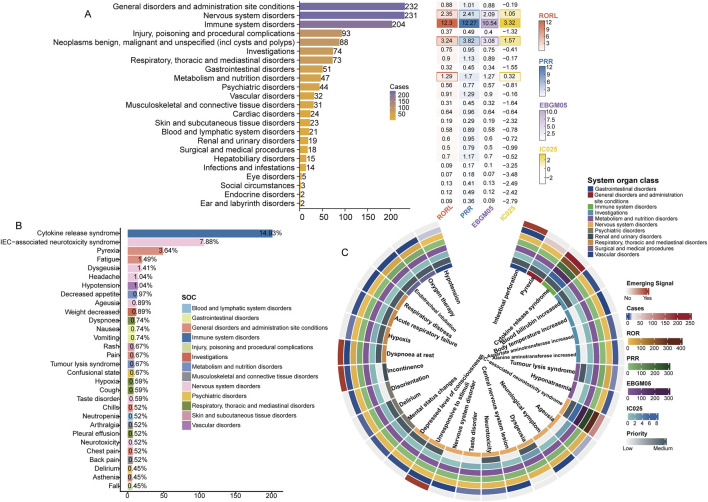
Disproportionality analysis results at the SOC and PT levels. **(A)** The bar chart on the left illustrates the distribution of adverse event reports associated with tarlatamab across System Organ Class (SOC) categories, while the heatmap on the right displays signal strength identified by four disproportionality analysis methods. SOCs meeting the predefined algorithm-specific thresholds are highlighted with dark borders. **(B)** The bar chart presents the top 30 preferred terms (PTs) ranked by the number of reported cases. Percentages indicate the proportion of each specific adverse event relative to all reported adverse events, with different colors representing their corresponding SOC classifications. **(C)** Signal detection at the PT level. The circular heatmap depicts the 30 PTs that met the positive signal thresholds across all four disproportionality algorithms, illustrating case counts, signal strength, clinical priority, and whether they were classified as emerging signals. The innermost ring indicates the SOC classification of each PT.

At the SOC level, the most frequently reported SOCs were General disorders and administration site conditions (n = 232), Nervous system disorders (n = 231), and Immune system disorders (n = 204). Using four disproportionality analysis methods, three SOCs met the predefined positive signal criteria across all algorithms (Supplementary Table S2). These were Nervous system disorders (ROR = 2.70, 95% CI: 2.35–3.11; PRR = 2.41; EBGM05 = 2.09; IC025 = 1.05), Immune system disorders (ROR = 14.28, 95% CI: 12.30–16.58; PRR = 12.27; EBGM05 = 10.54; IC025 = 3.32), and Neoplasms benign, malignant and unspecified (including cysts and polyps) (ROR = 4.02, 95% CI: 3.24–4.99; PRR = 3.82; EBGM05 = 3.08; IC025 = 1.57) (Figure 3A). In addition, Metabolism and nutrition disorders met the positive signal thresholds for the ROR and BCPNN methods (ROR = 1.73, 95% CI: 1.29–2.31; IC025 = 0.32), but not for PRR or MGPS.

#### 3.2.2 Signal detection at the PT level

At the PT level, the most frequently reported adverse events were CRS (n = 201, 14.93%), ICANS (n = 106, 7.88%), pyrexia (n = 49, 3.64%), fatigue (n = 20, 1.49%), dysgeusia (n = 19, 1.41%), headache (n = 14, 1.04%), hypotension (n = 14, 1.04%), decreased appetite (n = 13, 0.97%), ageusia (n = 12, 0.89%), and weight decreased (n = 12, 0.89%) (Figure 3B). A total of 30 PTs met the positive signal criteria across all four disproportionality analysis methods (Table 3). Frequently reported PTs with positive signals included CRS (n = 201; ROR = 223.84, 95% CI: 192.15–260.77; PRR = 190.56; EBGM05 = 156.70; IC025 = 6.36), ICANS (n = 106; ROR = 312.43, 95% CI: 254.68–383.26; PRR = 287.90; EBGM05 = 220.07; IC025 = 5.96), pyrexia (n = 49; ROR = 7.10, 95% CI: 5.34–9.44; PRR = 6.88; EBGM05 = 5.16; IC025 = 2.20), dysgeusia (n = 19; ROR = 20.45, 95% CI: 12.99–32.20; PRR = 20.18; EBGM05 = 12.76; IC025 = 2.71), hypotension (n = 14; ROR = 3.90, 95% CI: 2.31–6.61; PRR = 3.87; EBGM05 = 2.29; IC025 = 0.95), and ageusia (n = 12; ROR = 26.04, 95% CI: 14.73–46.05; PRR = 25.82; EBGM05 = 14.52; IC025 = 2.34). Several PTs not listed in the drug label also met the positive signal criteria, including incontinence (n = 4; ROR = 21.60, 95% CI: 8.08–57.78; PRR = 21.54; EBGM05 = 8.01; IC025 = 0.78), dyspnoea at rest (n = 3; ROR = 41.09, 95% CI: 13.16–128.24; PRR = 41.00; EBGM05 = 13.01; IC025 = 0.44), intestinal perforation (n = 3; ROR = 13.61, 95% CI: 4.38–42.32; PRR = 13.58; EBGM05 = 4.35; IC025 = 0.26), and unresponsive to stimuli (n = 3; ROR = 6.83, 95% CI: 2.20–21.22; PRR = 6.82; EBGM05 = 2.19; IC025 = 0.03). Several PTs with small numbers of reports showed high disproportionality estimates, including tumour lysis syndrome (TLS) (n = 9; ROR = 43.37, 95% CI: 22.44–83.81; PRR = 43.09; EBGM05 = 22.08; IC025 = 2.12), neurotoxicity (n = 7; ROR = 16.49, 95% CI: 7.84–34.71; PRR = 16.41; EBGM05 = 7.77; IC025 = 1.46), mental status changes (n = 5; ROR = 12.32, 95% CI: 5.11–29.70; PRR = 12.28; EBGM05 = 5.08; IC025 = 0.91), and central nervous system lesion (n = 3; ROR = 18.90, 95% CI: 6.07–58.81; PRR = 18.86; EBGM05 = 6.03; IC025 = 0.34) (Figure 3C).

**TABLE 3:** A total of 30 positive signals for tarlatamab meeting all four disproportionality thresholds are shown. Preferred terms (PTs) are presented in descending order by the number of cases.

SOC name	PT name	Case number	ROR (95% CI)	PRR (χ²)	EBGM (EBGM05)	IC(IC025)	Dead	Fatality rate
Cytokine release syndrome	Immune system disorders	201	223.84 (192.15–260.77)	190.56 (36330.39)	182.55 (156.7)	7.51 (6.36)	28	0.139303483
Immune effector cell-associated neurotoxicity syndrome	Nervous system disorders	106	312.43 (254.68–383.26)	287.9 (28420.04)	269.97 (220.07)	8.08 (5.96)	12	0.113207547
Pyrexia	General disorders and administration site conditions	49	7.1 (5.34–9.44)	6.88 (247.03)	6.87 (5.16)	2.78 (2.2)	2	0.040816327
Dysgeusia	Nervous system disorders	19	20.45 (12.99–32.2)	20.18 (344.99)	20.09 (12.76)	4.33 (2.71)	2	0.105263158
Hypotension	Vascular disorders	14	3.9 (2.31–6.61)	3.87 (29.91)	3.87 (2.29)	1.95 (0.95)	1	0.071428571
Ageusia	Nervous system disorders	12	26.04 (14.73–46.05)	25.82 (284.71)	25.67 (14.52)	4.68 (2.34)	0	0
Tumour lysis syndrome	Metabolism and nutrition disorders	9	43.37 (22.44–83.81)	43.09 (366.42)	42.67 (22.08)	5.42 (2.12)	4	0.444444444
Hypoxia	Respiratory, thoracic and mediastinal disorders	8	10.5 (5.23–21.05)	10.44 (68.17)	10.42 (5.19)	3.38 (1.38)	1	0.125
Taste disorder	Nervous system disorders	8	9.18 (4.58–18.41)	9.13 (57.85)	9.12 (4.55)	3.19 (1.29)	1	0.125
Neurotoxicity	Nervous system disorders	7	16.49 (7.84–34.71)	16.41 (100.95)	16.35 (7.77)	4.03 (1.46)	0	0
Delirium	Psychiatric disorders	6	7.65 (3.43–17.07)	7.62 (34.47)	7.61 (3.41)	2.93 (0.87)	0	0
Aspartate aminotransferase increased	Investigations	6	6.9 (3.09–15.39)	6.87 (30.07)	6.86 (3.08)	2.78 (0.81)	0	0
Hyponatraemia	Metabolism and nutrition disorders	5	5.35 (2.22–12.89)	5.34 (17.6)	5.33 (2.21)	2.41 (0.45)	0	0
Mental status changes	Psychiatric disorders	5	12.32 (5.11–29.7)	12.28 (51.69)	12.25 (5.08)	3.61 (0.91)	2	0.4
Alanine aminotransferase increased	Investigations	5	4.93 (2.05–11.86)	4.91 (15.58)	4.91 (2.04)	2.3 (0.39)	0	0
Incontinence^a^	Renal and urinary disorders	4	21.6 (8.08–57.78)	21.54 (77.96)	21.44 (8.01)	4.42 (0.78)	0	0
Disorientation	Psychiatric disorders	4	7.47 (2.8–19.96)	7.46 (22.33)	7.44 (2.79)	2.9 (0.41)	0	0
Acute respiratory failure	Respiratory, thoracic and mediastinal disorders	4	9.1 (3.41–24.31)	9.08 (28.7)	9.06 (3.39)	3.18 (0.5)	3	0.75
Depressed level of consciousness	Nervous system disorders	4	6.59 (2.47–17.61)	6.58 (18.9)	6.57 (2.46)	2.72 (0.34)	2	0.5
Blood bilirubin increased	Investigations	4	9.5 (3.56–25.38)	9.48 (30.27)	9.46 (3.54)	3.24 (0.52)	1	0.25
Oxygen therapy	Surgical and medical procedures	3	41.75 (13.37–130.32)	41.66 (117.91)	41.27 (13.22)	5.37 (0.44)	1	0.333333333
Dyspnoea at rest^a^	Respiratory, thoracic and mediastinal disorders	3	41.09 (13.16–128.24)	41 (115.97)	40.62 (13.01)	5.34 (0.44)	0	0
Endotracheal intubation	Surgical and medical procedures	3	47.91 (15.34–149.67)	47.81 (135.98)	47.29 (15.14)	5.56 (0.46)	1	0.333333333
Intestinal perforation^a^	Gastrointestinal disorders	3	13.61 (4.38–42.32)	13.58 (34.86)	13.54 (4.35)	3.76 (0.26)	0	0
Unresponsive to stimuli^a^	Nervous system disorders	3	6.83 (2.2–21.22)	6.82 (14.87)	6.81 (2.19)	2.77 (0.03)	1	0.333333333
Nervous system disorder	Nervous system disorders	3	8.14 (2.62–25.31)	8.13 (18.72)	8.11 (2.61)	3.02 (0.1)	0	0
Respiratory distress	Respiratory, thoracic and mediastinal disorders	3	6.73 (2.17–20.91)	6.72 (14.58)	6.71 (2.16)	2.75 (0.02)	1	0.333333333
Neurological symptom	Nervous system disorders	3	21.82 (7.01–67.94)	21.77 (59.17)	21.67 (6.96)	4.44 (0.36)	0	0
Body temperature increased	Investigations	3	8.04 (2.59–24.98)	8.02 (18.41)	8.01 (2.58)	3 (0.09)	0	0
Central nervous system lesion	Nervous system disorders	3	18.9 (6.07–58.81)	18.86 (50.52)	18.78 (6.03)	4.23 (0.34)	0	0

Fatality rate score	Reporting rate score	The number of algorithms that satisfy the positive signal value	The number of algorithms that satisfy the positive signal value	Signal stability score	Clinical relevance	Total score	Clinical priority
0	0.175545852	2	4	2	1	5	Medium
0	0.085483871	1	4	2	1	4	Medium
0	0.037779491	1	4	2	0	3	Medium
0	0.014318011	1	4	2	0	3	Medium
0	0.010510511	1	4	2	0	3	Medium
0	0.008995502	0	4	2	0	2	Low
1	0.006731488	0	4	2	1	4	Medium
0	0.005979073	0	4	2	1	3	Medium
0	0.005979073	0	4	2	0	2	Low
0	0.005227782	0	4	2	1	3	Medium
0	0.004477612	0	4	2	1	3	Medium
0	0.004477612	0	4	2	0	2	Low
0	0.003728561	0	4	2	0	2	Low
1	0.003728561	0	4	2	0	3	Medium
0	0.003728561	0	4	2	0	2	Low
0	0.002980626	0	4	2	0	2	Low
0	0.002980626	0	4	2	0	2	Low
2	0.002980626	0	4	2	1	5	Medium
1	0.002980626	0	4	2	1	4	Medium
1	0.002980626	0	4	2	0	3	Medium
1	0.002233805	0	4	2	0	3	Medium
0	0.002233805	0	4	2	1	3	Medium
1	0.002233805	0	4	2	0	3	Medium
0	0.002233805	0	4	2	2	4	Medium
1	0.002233805	0	4	2	1	4	Medium
0	0.002233805	0	4	2	0	2	Low
1	0.002233805	0	4	2	1	4	Medium
0	0.002233805	0	4	2	0	2	Low
0	0.002233805	0	4	2	0	2	Low
0	0.002233805	0	4	2	0	2	Low

^a^ Signals not described in the drug label are marked with asterisks.

#### 3.2.3 Clinical priority assessment

Based on the clinical priority scoring system, 19 of the 30 identified PT-level positive signals were classified as medium-priority signals, including CRS, ICANS, dysgeusia, hypotension, and TLS. The remaining 11 signals were classified as low-priority signals, including ageusia, taste disorder, and aspartate aminotransferase increased (Figure 3C).

### 3.3 Subgroup analysis

**FIGURE 4:**
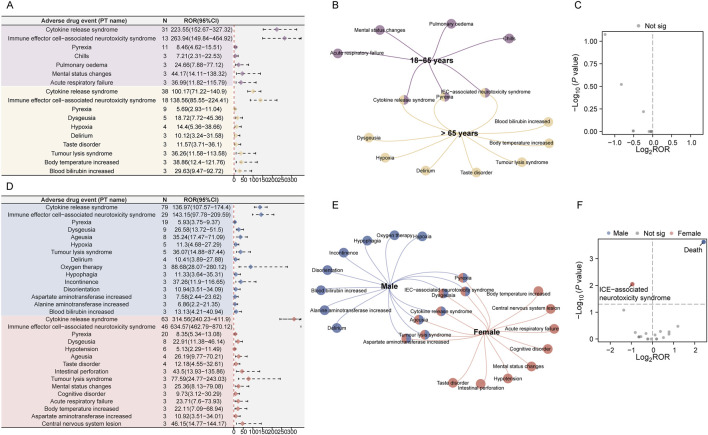
Subgroup analyses. **(A)** Age-based subgroup analysis. Forest plots illustrate all signals meeting the positive thresholds of four disproportionality analysis algorithms in the middle-aged group (18–65 years, purple background) and the older group (>65 years, orange background). The forest plot on the right presents the corresponding reporting odds ratios (RORs) with 95% confidence intervals. **(B)** Network Venn diagram depicting overlapping signals between age subgroups. **(C)** Volcano plots showing high-risk signals across different age subgroups. The x-axis represents the log2-transformed reporting odds ratio (log_2_ROR) calculated using the reporting odds ratio (ROR) method, and the y-axis represents the negative base-10 logarithm of the P value (−log10P) derived from the chi-square test. Each point corresponds to a preferred term (PT), with point size and color scaled according to its −log10P value. **(D)** Sex-based subgroup analysis. Forest plots display all signals meeting the positive thresholds of four disproportionality algorithms in male (blue background) and female (red background) subgroups, with the corresponding RORs and 95% confidence intervals shown on the right. **(E)** Network Venn diagram illustrating overlapping signals between sex subgroups. **(F)** Volcano plots depicting high-risk signals across sex-based subgroups.

To exclude potential confounding effects from demographic differences, we conducted stratified analyses based on age and sex. In the age subgroup analysis, 7 and 10 positive signals were identified in the middle-aged group (18–65 years) and older group (>65 years), respectively (Figure 4A). Overlapping positive signals included CRS, pyrexia, and ICANS (Figure 4B). Unique positive signals in the middle-aged group included mental status changes (ROR 44.17), acute respiratory failure (ROR 36.99), and others, while unique signals in the older group included dysgeusia (ROR 18.72), delirium (ROR 10.12), TLS (ROR 36.26), and blood bilirubin increased (ROR 29.63). However, after applying the adjusted ROR algorithm, no high-risk signals were identified in any specific subgroup (Figure 4C). In the sex subgroup analysis, 15 positive signals were identified in both the male and female subgroups (Figure 4D). Overlapping positive signals included CRS, dysgeusia, ICANS, and TLS. Unique positive signals in the male subgroup included hypophagia (ROR 11.33), incontinence (ROR 37.26), disorientation (ROR 10.94), blood bilirubin increased (ROR 13.13), and delirium (ROR 10.41), while unique signals in the female subgroup included central nervous system lesion (ROR 16.15), cognitive disorder (ROR 9.73), hypotension (ROR 5.13), intestinal perforation (ROR 43.5), and taste disorder (ROR 12.18) (Figure 4E). After applying the adjusted ROR algorithm, high-risk signals identified in the male subgroup included death, while in the female subgroup, the high-risk signal identified was ICANS (Figure 4F).

### 3.4 Results of machine learning model performance evaluation

**FIGURE 5:**
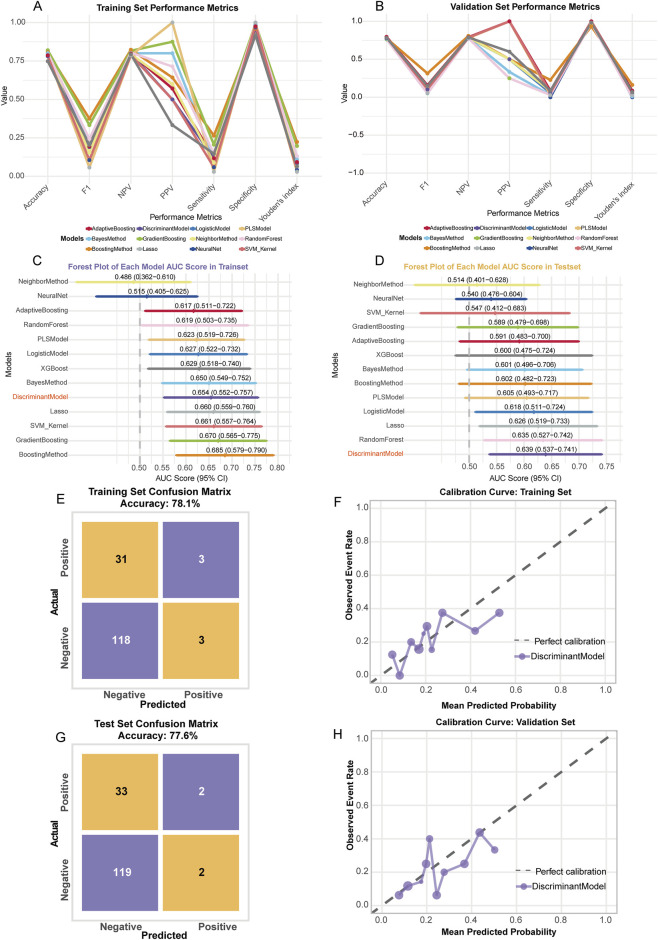
Development and evaluation of 13 machine learning models. **(A,B)** show the prediction performance metrics in the training and test models, respectively. **(C,D)** present the forest plots of AUC values in the training and test models, respectively. **(E,G)** display the confusion matrices of the best model (discriminant model) in the training and test sets. **(F,H)** show the calibration curves of the best model (discriminant model) in the training and test sets. PPV, positive predictive value; NPV, negative predictive value.

A total of 13 MLs algorithms were developed to construct binary classification models for identifying report-level patterns associated with tarlatamab-related immune adverse events. Model performance was evaluated using multiple metrics, including accuracy, F1 score, NPV, PPV, sensitivity, specificity, and Youden’s index, in both the training and validation datasets (Supplementary Table S3). As shown in Figure 5A, model performance varied across algorithms in the training set. Overall, ensemble-based methods, including Gradient Boosting, XGBoost, Random Forest, and Boosting-based models, demonstrated relatively balanced performance across most metrics, particularly in terms of PPV, specificity, and Youden’s index. In contrast, simpler linear models and distance-based methods showed lower sensitivity and F1 scores. Consistent patterns were observed in the validation set (Figure 5B), where ensemble learning models generally maintained stable performance. Although sensitivity values declined across most models in the validation cohort, specificity remained high, indicating a tendency toward conservative prediction of immune adverse events. Receiver operating characteristic (ROC) curve analysis further compared the discriminative ability of each model. In the training set (Figure 5C), the area under the ROC curve (AUC) ranged from 0.486 to 0.685. Among all models, the Boosting-based method achieved the highest AUC (0.685, 95% CI: 0.579–0.790), followed by Gradient Boosting (AUC = 0.670, 95% CI: 0.565–0.775), SVM with kernel function (AUC = 0.661, 95% CI: 0.557–0.764), and the Discriminant Model (AUC = 0.654, 95% CI: 0.552–0.757). In the validation set (Figure 5D), the AUC values ranged from 0.514 to 0.639. The Discriminant Model showed the highest AUC (0.639, 95% CI: 0.537–0.741), followed by Random Forest (AUC = 0.635, 95% CI: 0.527–0.742), Lasso regression (AUC = 0.626, 95% CI: 0.519–0.733), and Logistic Regression (AUC = 0.618, 95% CI: 0.511–0.724), indicating comparatively better generalization performance. Given its balanced discrimination ability and stable performance across datasets, the Discriminant Model was selected as the optimal model for further evaluation. The confusion matrix of the training set (Figure 5E) showed an overall accuracy of 78.1%, while the validation set achieved an accuracy of 77.6% (Figure 5G), indicating consistent classification performance between datasets. Calibration analysis demonstrated reasonable agreement between predicted probabilities and observed outcomes. In the training set (Figure 5F), the calibration curve of the Discriminant Model showed moderate deviation from the ideal reference line. Similar calibration patterns were observed in the validation set (Figure 5H), suggesting acceptable calibration performance of the model across different data splits.

### 3.5 SHAP explainability analysis

**FIGURE 6:**
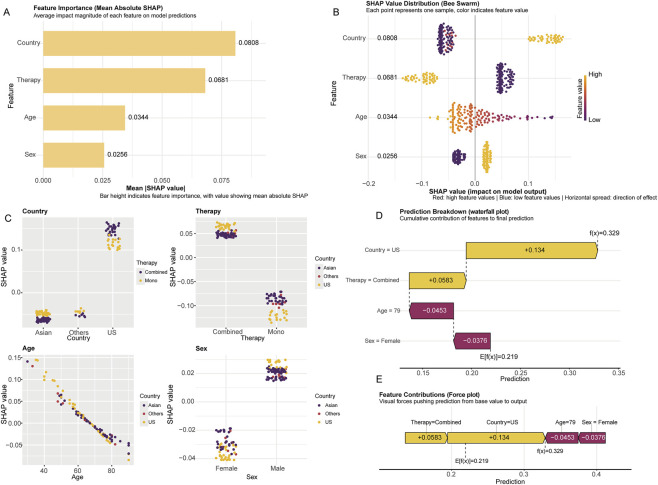
SHAP value interpretation of features in the discriminant model. **(A)** Bar chart showing the contribution of four features in the discriminant model. **(B)** Beeswarm plot: Each point represents the SHAP value of a feature for an individual sample. Features are vertically ordered by global importance, from highest (top) to lowest (bottom). The horizontal axis displays SHAP values, where positive values (right) indicate contributions to a higher predicted risk class, and negative values (left) indicate contributions to a lower risk class. Color intensity reflects feature magnitude (yellow: high, purple: low). **(C)** Scatter plot illustrating the correlation between different features and their SHAP values. **(D)** Waterfall plot and **(E)** heatmap showing the SHAP value contributions of each feature to a single prediction.

The SHAP values were used to interpret the importance and contribution of each feature in the optimal model. The feature importance analysis (Figure 6A) revealed that the most influential feature was country (mean absolute SHAP = 0.0808), followed by therapy type (mean absolute SHAP = 0.0681), age (mean absolute SHAP = 0.0344), and sex (mean absolute SHAP = 0.0256). The distribution of SHAP values (Figure 6B) indicated that the country feature exhibited a strong influence on the model’s predictions, with higher feature values (indicating stronger impacts on the prediction) associated with reports from the US, while Asian and other countries had lower feature values. Therapy also had a notable impact, with the combined therapy showing higher positive SHAP values compared to mono therapy. Age demonstrated a consistent negative influence on the model’s output, with older age groups contributing to lower predictions, as shown by the negative SHAP values in the age plot (Figure 6C). To better illustrate the contribution of features to model predictions and the prediction process, we employed the shapviz package to generate a breakdown plot and a force plot. The prediction breakdown (Figure 6D) demonstrated how each feature contributed to single model prediction. The Country feature, with the highest positive SHAP value of +0.134, had the largest contribution, followed by therapy type (combined therapy vs. mono) with +0.0583. The age feature contributed negatively to the prediction, with an SHAP value of −0.0453 for an age of 79 years. Sex also had a negative contribution, with female reports contributing −0.0376 to the prediction. The final predicted probability was 0.329, calculated based on the cumulative contribution of all features. The feature contributions (Figure 6E) further illustrated the forces pushing the prediction from the base value (0.219) to the final output (0.329). The therapy type = combined and country = US had the strongest positive effects, while age = 79 and sex = Female exerted negative contributions. The model’s output was primarily influenced by the interaction between therapy type and geographical location, with age and sex playing secondary roles, indicating that prediction was driven more by reporting context and treatment scenario than by variables plausibly reflecting intrinsic patient susceptibility.

### 3.6 Logistic regression analysis to identify high-risk factors for tarlatamab-associated cytokine release syndrome

**FIGURE 7:**
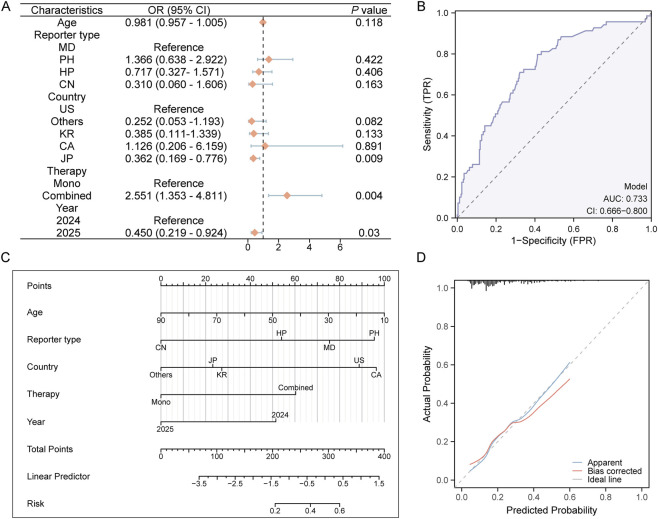
Multivariable logistic regression analysis identifying high-risk factors for tarlatamab-related cytokine release syndrome. **(A)** Forest plot showing adjusted odds ratios (aORs) with 95% confidence intervals (CIs) for variables independently associated with tarlatamab-related cytokine release syndrome. **(B)** Receiver operating characteristic (ROC) curve evaluating the discriminative performance of the multivariable model. **(C)** Nomogram constructed to estimate the risk of tarlatamab-related cytokine release syndrome. **(D)** Calibration curve assessing agreement between predicted probabilities and observed outcomes. TPR, true positive rate; FPR, false positive rate; MD, physician; PH, pharmacist; HP, health professional; CN, consumer; US, United States; KR, Korean; CA, Canada; JP, Japan.

In both overall and stratified analyses, CRS was consistently identified as a stable positive signal, indicating a strong potential association with tarlatamab use. To further evaluate the relationship between tarlatamab and CRS under different confounding factors, univariate and multivariate logistic regression analyses were performed. The univariate analysis revealed that age (OR = 0.972, 95% CI: 0.952–0.993, *P* = 0.010), reports from Japan (OR = 0.336, 95% CI: 0.202–0.557, *P* < 0.001), reports from other regions (OR = 0.140, 95% CI: 0.033–0.583, *P* = 0.007), and reports from 2025 (OR = 0.228, 95% CI: 0.164–0.316, *P* < 0.001) were protective factors for tarlatamab-related CRS, while reports from pharmacists (OR = 2.342, 95% CI: 1.554–3.532, *P* < 0.001) and reports with concomitant medications (OR = 1.625, 95% CI: 1.184–2.230, *P* = 0.003) were risk factors (Supplementary Table S4). Multivariate logistic regression analysis confirmed that reports from Japan (OR = 0.336, 95% CI: 0.202–0.557, *P* < 0.001) and year of 2025 (OR = 0.450, 95% CI: 0.219–0.924, *P* = 0.03) were protective factors, while reports with concomitant medications (OR = 2.551, 95% CI: 1.353–4.811, *P* = 0.004) remained a significant risk factor for tarlatamab-related CRS (Figure 7A). The adjusted multivariate logistic regression model demonstrated good predictive performance, with AUC = 0.733 (Figure 7B), indicating the model’s capacity for discrimination and prediction. Based on the multivariate logistic regression model, a nomogram was developed to predict the risk of CRS following tarlatamab use (Figure 7C). Further validation through a calibration plot demonstrated minimal deviation from the ideal line, confirming the accuracy and utility of the model in clinical prediction of tarlatamab-related CRS (Figure 7D).

Additionally, risk factors for CRS-related mortality were identified. While univariate logistic regression analysis showed associations with gender, reporter type, and country of report, multivariate logistic regression identified that female reporters were at higher risk of CRS-related mortality compared to male reporters (OR = 4.741, 95% CI: 1.143–19.664, *P* = 0.032) (Supplementary Table S5). The multivariate logistic regression model for CRS-related mortality demonstrated an AUC of 0.830 (Supplementary Figure S1A). Nomogram and calibration plots for this model were also developed (Supplementary Figures S1B and C).

In the above analysis, concomitant medication use was statistically associated with CRS reports involving tarlatamab. To further elucidate the specific co-medications reported in these CRS cases, we summarized the ten most frequently reported concomitant drugs (Supplementary Figure S2). The top five were dexamethasone (n = 20), tocilizumab (n = 8), oxycodone (n = 6), sodium chloride (n = 5), and acetaminophen (n = 4).

### 3.7 Analysis of the timing to onset of ADEs

**FIGURE 8:**
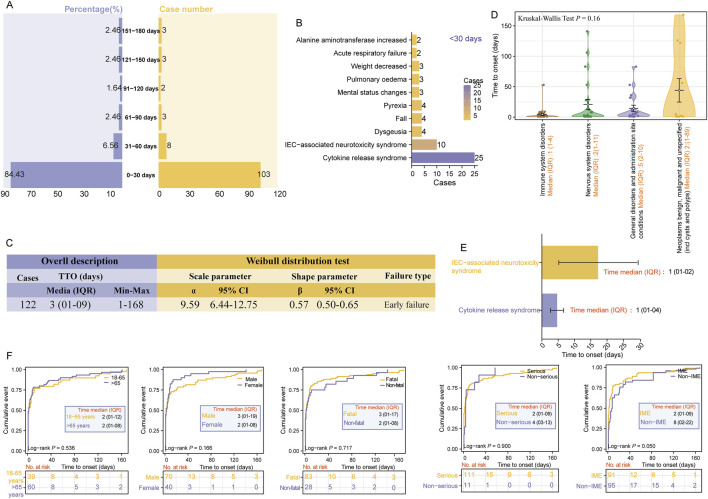
Time-to-onset (TTO) analysis of tarlatamab-related adverse drug events (ADEs). **(A)** Bar charts showing the number and proportion of TTO reports across different time intervals. **(B)** Top 10 PTs with adverse events occurring within less than 30 days were ranked according to the number of reported cases. **(C)** The Weibull distribution analysis results fitting for TTO assessment. **(D)** Box plots illustrating TTO distributions of ADEs at the System Organ Class (SOC) level. **(E)** The bar chart compares the differences in the occurrence times of adverse events between ICANS and cytokine release syndrome. **(F)** Cumulative distribution curves were generated to compare TTO variations according to age, sex, fatality outcome (fatal vs. non-fatal), outcome severity (serious vs. non-serious), and whether it is an important medical event (IME vs. non-IME). IQR, interquartile range; Min, minimum; Max, maximum. IME, important medical event; IEC, immune effector cell.

A total of 122 reports containing information on the timing of adverse events were collected from the FAERS database. Most of these events occurred within 1 month of drug use (n = 103, 84.43%), with a smaller proportion reported in the second month (n = 8, 6.56%) (Figure 8A). At the PT level, the three most common adverse events occurring within 30 days were CRS (n = 25), ICANS (n = 10), and dysgeusia (n = 4) (Figure 8B). The median time to onset of all adverse events was 3 days. Weibull distribution analysis showed a shape parameter β of 0.57, with an upper 95% confidence interval of 0.65, indicating early decay in onset times (Figure 8C). At the SOC level, among the four SOCs with more than 10 reports, immune system disorders had the shortest median onset time (1 day), while general disorders and administration site conditions had the longest median onset time (5 days). However, no significant differences in onset time were observed across SOCs (Kruskal–Wallis test, *P* = 0.16) (Figure 8D). At the PT level, both CRS and ICANS had a median onset time of 1 day (Figure 8E). Subgroup analysis revealed no significant differences in the cumulative incidence of adverse events based on age (18–65 years vs. >65 years, log-rank *P* = 0.539), sex (male vs. female, log-rank *P* = 0.166), death outcome (death vs. non-death, log-rank *P* = 0.717), or severity (severe vs. non-severe, log-rank *P* = 0.900). However, in the serious medical events subgroup, the median onset time for serious events (2 days) was earlier than for non-serious events (8 days), with a log-rank test result of *P* = 0.05 (Figure 8F).

### 3.8 Cross-descriptive comparison with the WHO-VigiAccess

**FIGURE 9:**
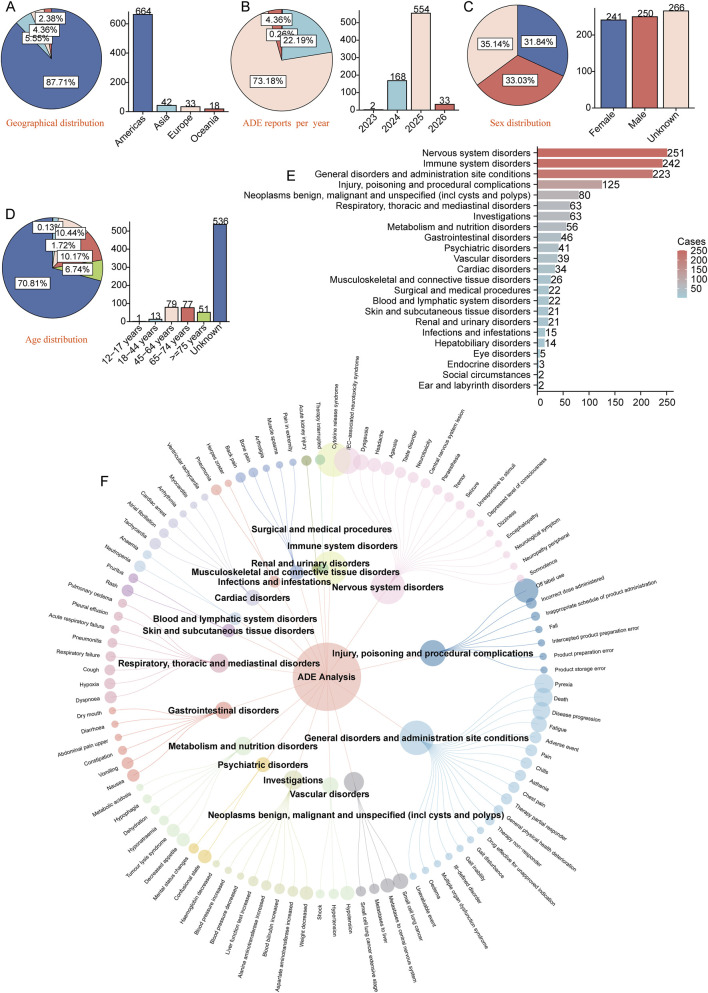
Descriptive analysis from WHO-VigiAccess: Clinical characteristics of ADE reports associated with tarlatamab. **(A)** Geographical distribution. **(B)** Adverse drug event (ADE) reports per year. **(C)** Sex distribution. **(D)** Age distribution. **(E)** Number of ADE reports at the SOC level. **(F)** Circular network plot showing the SOC distribution (color-coded circles) and case count (circle size) for the top 100 ADE reports ranked by number of cases. SOC, system organ class.

A total of 757 ICSRs related to tarlatamab were retrieved from the WHO-VigiAccess database, comprising 1,604 reported ADEs. Similar to the FAERS database, most reports originated from the Americas (n = 664, 87.71%), followed by Asia, Europe, and Oceania (Figure 9A). The annual distribution of reports showed a peak in 2025, with 554 reports, accounting for 73.18% of all cases (Figure 9B). Reports were similarly distributed between males (31.84%) and females (33.03%), while sex information was missing in the remaining cases (Figure 9C). Although age information was unavailable in 70.81% of the reports, among cases with known age, the highest proportions were observed in patients aged 45–65 years (10.44%), 65–74 years (10.17%), and ≥75 years (6.74%) (Figure 9D). Overall, the reported adverse events spanned 23 SOCs. The three most frequently reported SOCs were Nervous system disorders (n = 251), Immune system disorders (n = 242), and General disorders and administration site conditions (n = 223) (Figure 9E). At the PT level, a total of 377 distinct PTs was identified. Figure 9F presents the top 100 most frequently reported PTs and their corresponding SOC classifications. The five most commonly reported PTs were CRS (n = 241), ICANS (n = 128), off-label use (n = 97), pyrexia (n = 49), and death (n = 47) (Figure 9F).

## 4 Discussion

Lung cancer is one of the most common malignancies worldwide and remains a leading cause of cancer-related mortality (Zhang et al., 2023). SCLC is characterized by rapid proliferation, an early propensity for metastasis, and low immunogenicity, resulting in a poorer prognosis compared with many other solid tumors. Although patients with SCLC are typically sensitive to initial chemotherapy, most eventually experience disease progression within 1 year due to the development of acquired resistance (Jin et al., 2023). BiTE therapy has emerged as a promising immunotherapeutic strategy in the treatment of various malignancies. Its mechanism involves the generation of a bispecific antibody capable of simultaneously recognizing and binding both T cells and tumor cells, thereby directing T cells into close proximity with malignant cells and activating their cytotoxic function. This process enables efficient tumor cell elimination while minimizing damage to normal tissues (Tang and Kang, 2023). Tarlatamab, which operates through this unique mechanism, provides an important therapeutic option for patients with SCLC. In the present study, we conducted a comprehensive safety evaluation of tarlatamab using real-world data from the FAERS and WHO-VigiAccess databases. Our aim was to systematically assess the benefit–risk profile of this agent and provide evidence-based insights to support its rational use in clinical practice.

### 4.1 Baseline characteristics of FAERS reports involving tarlatamab

The overall reporting pattern was broadly consistent with the clinical context of tarlatamab use in relapsed ES-SCLC (Dhillon, 2024). Reports increased markedly in 2025 relative to 2024, likely reflecting expanding post-approval use and growing familiarity with this agent in routine practice (Mountzios et al., 2025). However, this increase should be interpreted cautiously, because spontaneous reporting system may also be influenced by stimulated reporting, market uptake, and time-related reporting behaviors rather than by a proportional increase in the true frequency of ADEs (Arora et al., 2017). The predominance of reports in older adults and the higher proportion of male reports were also generally compatible with the demographic profile of the SCLC population (Bray et al., 2024; Rossi et al., 2005; Gironés et al., 2011). In addition, most reports were submitted by physicians and other healthcare professionals, which may support the clinical relevance of the reported events. At the same time, several baseline variables were incompletely captured, particularly body weight and concomitant medication information, which limited more detailed subgroup interpretation. Overall, these baseline findings are useful for contextualizing the reporting population but should not be interpreted as direct estimates of exposure distribution or real-world event incidence.

### 4.2 Disproportionality-based safety signals of tarlatamab

At both the SOC and PT levels, the disproportionality pattern was broadly consistent with the established safety profile of tarlatamab, supporting the face validity of the present analysis.

The most prominent PT-level signals, particularly CRS, ICANS, pyrexia, dysgeusia, hypotension, and ageusia, were generally concordant with toxicities already recognized in clinical trials and clinical management reports (Ahn et al., 2023; Sands et al., 2025). This concordance strengthens the face validity of the current pharmacovigilance results. Studies have shown that CRS may occur following treatment with tarlatamab. The onset of CRS stems from the massive release of various pro-inflammatory cytokines, including IL-6, TNF-α and IFN-γ, by B cells, T cells and NK cells. These factors further activate cells such as endothelial cells, monocytes/macrophages and dendritic cells; the latter, in turn, accelerate the excessive secretion of cytokines, forming a cascading inflammatory loop that ultimately leads to endothelial dysfunction and capillary leakage, thereby exacerbating clinical symptoms. ICANS refers to a clinical syndrome in which, following any form of immunotherapy, endogenous or infused T cells and other immune effector cells are activated and become involved, thereby triggering pathological processes in the central nervous system. Symptoms and signs may worsen progressively, with clinical manifestations including aphasia, decreased level of consciousness, cognitive impairment, motor weakness, seizures and cerebral oedema (Lee et al., 2019). DLL3 is expressed in the brain and pituitary gland, albeit at low levels (Le et al., 2025). Upon binding to this receptor, the drug tarlatamab may further induce CRS, leading to increased blood-brain barrier permeability, allowing activated T cells and cytokines to enter the central nervous system (Gust et al., 2017). Research indicates that interleukin-6 (IL-6) exerts significant effects within the nervous system, and its abnormal expression has been shown to contribute to the pathological processes of various neurological disorders. These include Alzheimer’s disease, Parkinson’s disease, schizophrenia and depression (Lee et al., 2014). Clinical pathological studies have shown that lymphocytic infiltration around blood vessels is observed in patients presenting with severe neurological symptoms. Such patients subsequently develop a series of neurological adverse symptoms (severe cases may present with epilepsy, cerebral oedema, *etc.*) (Gust et al., 2017). The Sixth International Consultative Committee on Urinary Incontinence has identified the following factors as clear risk factors for male urinary incontinence: advanced age, lower urinary tract symptoms, urinary tract infections, functional and cognitive impairment, diabetes, alcohol consumption, neurological disorders, and prostatectomy (Olagundoye et al., 2023). Consequently, when neurotoxicity occurs, vigilance regarding the onset of incontinence symptoms may be warranted. In severe cases, coma may even develop, leading to a lack of response to stimuli. The presence of the aforementioned signs should be given due attention in clinical practice; it is recommended that monitoring of patients’ post-medication status be intensified during the early stages of treatment, and that their clinical significance be further validated and explored in future research.

TLS and several less-recognized serious signals, including intestinal perforation and dyspnoea at rest, also merit attention. Although the absolute number of reports was limited, they are clinically important because they may represent severe toxicity scenarios in a highly vulnerable ES-SCLC population. In particular, dyspnoea at rest may arise in the context of severe systemic inflammation, hypoxemia, pulmonary capillary leak, infection, tumor progression, or decompensation of baseline thoracic disease (Shimabukuro-Vornhagen et al., 2018), whereas intestinal perforation is more cautiously interpreted as a potentially multifactorial event related to inflammatory injury, ischemia, infection, corticosteroid exposure, bowel fragility, or tumor-related gastrointestinal vulnerability rather than a direct drug-specific effect alone (Kido et al., 2022). Likewise, TLS is biologically and clinically plausible in patients with high tumor burden and treatment-sensitive disease, even though the number of reports was limited (Lobsiger et al., 2026; Mughal et al., 2010). Dysgeusia and ageusia were both statistically significant in terms of PT rankings and signal values in this study. Studies have shown that inflammation-induced changes in the interferon and Toll-like receptor pathways inhibit the normal renewal process of taste bud cells. Furthermore, the inflammatory response triggered by lipopolysaccharides not only reduces the proliferative capacity of taste progenitor cells but also accelerates the apoptosis of taste bud cells (Schalk et al., 2018). During treatment with tarlatamab, large amounts of inflammatory factors such as interferon are released, which may be the cause of this adverse reaction. In cancer patients, anorexia is a common symptom due to their treatment and underlying conditions (Schalk et al., 2018). This leads to a reduced appetite in patients, which in turn affects their physical performance scores and tolerance of subsequent treatment.

Among the detected signals, CRS (n = 201, ROR = 223.84, 95% CI: 192.15–260.77; PRR = 190.56; EBGM05 = 156.7; IC025 = 6.36) and ICANS (n = 106, ROR = 312.43, 95% CI: 254.68–383.26; PRR = 287.9; EBGM05 = 220.07; IC025 = 5.96) showed medium clinical priority. The occurrence of these two ADEs may pose a life-threatening risk. Studies have confirmed that the pre-treatment prophylactic use of tocilizumab with the T-cell engager teclistamab for multiple myeloma significantly reduces the incidence of immune-related adverse events. In existing studies, the incidence of CRS has been reduced from 89% to an average of 20%, whilst the incidence of ICANS has fallen from 20% to 5.3% (Zhang et al., 2026). Data indicate that elevated serum lactate dehydrogenase (LDH) levels, the presence of liver metastases, and a history of diabetes and cardiovascular disease are independent predictors of CRS and ICANS, respectively (Joshi et al., 2026). Overall, these findings help prioritize the main immune-mediated toxicities of tarlatamab for monitoring in routine practice, while less frequent signals should be interpreted as clinically cautionary but hypothesis-generating observations.

### 4.3 Subgroup differences in disproportionality signals

Subgroup analyses showed that the major disproportionality signals of tarlatamab remained broadly consistent across age and sex strata, with CRS and ICANS recurring as common signals in multiple subgroups, suggesting that the principal safety profile was largely stable across demographic categories (Ahn et al., 2023; Paz-Ares et al., 2023; Sands et al., 2025). Signals specific to the elderly group included dysgeusia (ROR 18.72), delirium (ROR 10.12), TLS (ROR 36.26), and elevated blood bilirubin. The middle-aged group, meanwhile, exhibited mental status changes (ROR 44.17) and acute respiratory failure (RLR 36.99), amongst others. Although some subgroup-specific signals were observed, particularly in older adults, these patterns may reflect a combination of reduced physiologic reserve, greater comorbidity burden, treatment complexity, and reporting heterogeneity rather than clear subgroup-specific toxicity mechanisms (Sodal et al., 2021; Alvis and Hughes, 2015; Abudahab et al., 2024). This suggests that when administering tarlatamab to elderly SCLC patients, clinicians should intensify monitoring of these parameters, given the potential decline in hepatic and renal reserve capacity and reduced ability to regulate electrolytes.

In the sex-stratified analysis, ADEs specific to males include loss of appetite (ROR 11.33), incontinence (ROR 37.26), disorientation (ROR 10.94), elevated serum bilirubin (ROR 13.13) and delirium (ROR 10.41). ADEs specific to females include central nervous system disorders (ROR 16.15), cognitive impairment (ROR 9.73), hypotension (ROR 5.13), intestinal perforation (ROR 43.5) and taste disturbances (ROR 12.18). The above data indicate that there is indeed a differentiated risk profile among different populations following treatment with tarlatamab. The higher incidence of incontinence in the male subgroup may be attributed to the fact that patients with indwelling catheters are classified as incontinent, which may have inflated the prevalence of incontinence (Condon et al., 2019). Results from a CRS risk model incorporating data from over 100 patients suggest that risk factors associated with the development of CRS may include female gender, liver metastases, and a number of metastatic sites exceeding three (Zhang et al., 2026). Studies have shown that the clinical manifestations of ICANS typically follow CRS, suggesting that CRS may act as an initiating factor or a contributing factor in the pathological cascade of ICANS(Morris et al., 2022). Consequently, females are more prone to intestinal perforation and central nervous system complications. Overall, these subgroup analyses are best interpreted as exploratory and hypothesis-generating. They may be useful for clinical vigilance and for prioritizing future validation studies, but they should not be overinterpreted as evidence of definitive demographic risk stratification.

### 4.4 Predictive modeling of immune-related toxicity and CRS associated with tarlatamab

Beyond disproportionality analysis, we explored whether report-level characteristics could help classify tarlatamab-related immune disorders. Overall, the ML results suggested that such an approach may be useful for pattern recognition and signal enrichment within spontaneous reporting data, although the discriminative performance of the tested models remained modest. Among the 13 algorithms evaluated, the discriminant model showed the most stable performance across the training and validation cohorts, with relatively consistent accuracy and acceptable calibration (Alba et al., 2017). Given the validation AUC of 0.639, however the model should not be interpreted as a bedside clinical risk prediction tool. Rather, its main value lies in providing an interpretable framework for stratifying report-level patterns and highlighting contexts in which immune-related reports may be more concentrated.

The SHAP analysis further clarified that the most influential features were country and therapy type, whereas age and sex made smaller contributions. This pattern suggests that model output was driven more by reporting context, treatment complexity, market uptake, and healthcare-system/reporting practices than by variables plausibly reflecting intrinsic biological susceptibility. Accordingly, the SHAP findings are better understood as describing how the model stratifies spontaneous reporting patterns than as identifying clinically actionable patient-level predictors (Ali et al., 2023).

Against this background, it was clinically reasonable to further focus on CRS as a specific endpoint for dedicated risk modeling. In our study, CRS was the most stable immune-related signal across the overall, subgroup, and disproportionality analyses, which is consistent with the established clinical safety profile of tarlatamab and other immune effector therapies. Recent multi-institutional real-world experience has likewise emphasized that CRS and ICANS remain the principal practical toxicities shaping tarlatamab administration and monitoring pathways, reinforcing the clinical relevance of building a CRS-focused analytic model (Zhang et al., 2026).

Compared with the broader machine-learning framework, the multivariable logistic regression model for CRS provided a more transparent and clinically interpretable prediction structure. Reports from Japan and those submitted in 2025 were associated with lower reported odds of CRS, whereas concomitant medication use was associated with higher reported odds. However, these variables should be interpreted cautiously, because in a spontaneous reporting system they are more likely to reflect differences in treatment environment, supportive care, patient selection, and reporting behavior than direct biologic protection or susceptibility. This concern is especially important for concomitant medication use. In our supplementary summary of co-medications among CRS cases, dexamethasone and tocilizumab were among the most frequently reported drugs. Because these agents are commonly administered as rescue therapies after CRS onset in routine clinical practice, the observed association between concomitant medication use and CRS is highly vulnerable to indication bias and reverse causality. Therefore, this variable should not be interpreted as evidence that co-medications increased the antecedent risk of CRS. rather, it more likely reflects post-event treatment, case severity, supportive care intensity, or broader reporting context. In this regard, the logistic regression findings are directionally consistent with the SHAP results, in that both analyses suggest that reporting context and treatment complexity substantially shape model output.

Notably, the logistic model achieved desirable discrimination for CRS prediction, and the corresponding nomogram showed acceptable calibration. Even so, this result should not be overstated. As with the machine-learning model, the CRS nomogram is better understood as an exploratory supportive tool for structured report-level risk awareness within pharmacovigilance analyses rather than as a stand-alone bedside clinical instrument. Its function is to help identify reports or patient contexts that may warrant closer review, especially during early treatment cycles or in more complex treatment settings, rather than to serve as a substitute for clinical judgment (Alba et al., 2017; Huang et al., 2020).

The analysis of CRS-related death requires even greater caution. Although female sex remained associated with higher reported odds of fatal CRS outcome in the multivariable model, the small number of events and wide confidence interval indicate substantial uncertainty. At the same time, this observation should not be dismissed outright. A scoping review of pharmacovigilance databases has suggested that sex-related differences in adverse drug reaction reporting are common, and that female may be overrepresented in spontaneous reports partly because of greater attention to health status, symptom perception, and reporting behavior (Brabete et al., 2022). Such mechanisms may contribute to the higher female signal observed in our model and could partly explain why sex emerges in report-based analyses even when biological causality remains uncertain. However, the same literature also supports the possibility that sex-related biological differences in drug response and toxicity may exist, meaning that the observed association between female sex and CRS-related fatal outcome may reflect both reporting propensity and true vulnerability, which cannot be disentangled in FAERS alone.

Taken together, the machine-learning and logistic regression analyses suggest that interpretable modeling can complement disproportionality analysis by improving the stratification of report patterns and interpretability of tarlatamab-related immune disorders signals, particularly for CRS. However, there models remain fundamentally constrained by the structure covariates, external prospective datasets and formal validation will be necessary before any such approaches can be considered for patient-level clinical prediction (Dimitsaki et al., 2024; Algarvio et al., 2025).

### 4.5 Temporal characteristics of tarlatamab-associated ADEs

The TTO analysis adds an important clinical dimension to the safety profile of tarlatamab by showing that reported adverse events clustered early after treatment initiation, with CRS and ICANS appearing particularly early. This pattern is consistent with existing trial and real-world experience indicating that the principal immune-mediated toxicities of tarlatamab emerge predominantly during the initial treatment period (Ahn et al., 2023; Sands et al., 2025). The Weibull analysis was likewise compatible with an early-onset reporting profile rather than a delayed cumulative toxicity pattern. Together with our earlier findings that CRS and ICANS were among the most prominent safety signals, these temporal data reinforce the view that the principal safety challenge of tarlatamab lies in the early recognition and management of acute immune-mediated toxicities, rather than delayed cumulative toxicity. This interpretation is further supported by emerging real-world evidence showing that higher rates of CRS and ICANS were observed during the first treatment cycle, again highlighting the importance of vigilance during the earliest administrations (Bolte et al., 2025). However, the absence of significant onset differences across most demographic and outcome-based subgroups suggests that this early-occurring pattern may be a broadly shared feature of tarlatamab-related toxicity rather than one confined to specific patient subsets. At the same time, because FAERS onset analyses are inherently limited by incomplete date recording, selective reporting, and the lack of denominator information, these findings should be interpreted cautiously because the TTO analysis was limited to reports with usable date information and, in the absence of reliable exposure denominators, cannot be used to estimate time-dependent incidence or hazard. Prospective cohorts with structured longitudinal follow-up will be necessary to validate these temporal patterns (Jin et al., 2026).

### 4.6 Cross-database descriptive comparison using the WHO VigiAccess

The WHO VigiAccess analysis provided across-database descriptive comparison for our FAERS-based findings. Despite differences in database structure and analytic depth, the overall reporting pattern was highly consistent across the two sources, with reports concentrated in the Americas and predominantly involving nervous system disorders, immune system disorders, and general disorders and administration site conditions. At the PT level, CRS and ICANS remained the most frequently reported toxicities, in line with the established clinical and real-world safety profile of tarlatamab. This cross-database concordance supports the consistency of the overall descriptive reporting pattern observed in FAERS, particularly the prominence of early immune-mediated and neurologic adverse events. However, this comparison should not be interpreted as independent external validation, because VigiBase, the underlying database of WHO-VigiAccess, may include reports originating from FAERS and other overlapping national pharmacovigilance systems. Therefore, the two data sources are not fully independent. In addition, because VigiAccess is primarily a global descriptive signal resource and contains substantial missing demographic information, it should be interpreted mainly as a tool for pattern corroboration rather than causal inference or quantitative risk estimation, or formal validation. Taken together, the WHO findings serve as a descriptive cross-database pattern corroboration of our FAERS analyses and further support the conclusion that the principal post-marketing safety challenge of tarlatamab lies in the recognition and management of CRS/ICANS-centered immune toxicity, while prospective real-world studies remain necessary to define incidence and patient-level risk more precisely (Jin et al., 2026; Wang et al., 2026).

### 4.7 Limits

This study has several important limitations that should be considered when interpreting the findings. First, as a spontaneous reporting system, FAERS does not provide a reliable denominator for drug exposure; therefore, incidence rates, absolute risks, and comparative risks cannot be estimated from these data. Disproportionality metrics reflect reporting frequency relative to a reference background rather than true event probability (Liu et al., 2025). Second, FAERS is subject to multiple forms of reporting bias and residual confounding, including under-reporting, stimulated reporting after drug approval or heightened clinical attention, differential reporting across countries and reporter types, and incomplete capture of disease severity, comorbidities, concomitant medications, prior anticancer therapies, and supportive care practices. As a result, observed signals and report-level associations may reflect a mixture of drug effects, patient vulnerability, treatment context, and reporting behavior. Third, missing data remained substantial for several variables, particularly body weight and concomitant medication information. Because body weight was unavailable in more than 90% of reports, it could not be reliably incorporated into subgroup analyses or model development. Likewise, regression and machine-learning analyses were based on complete-case subsets, which may have introduced selection bias and limited generalizability (Hu et al., 2025). Fourth, the machine-learning and SHAP analyses should be interpreted as exploratory and pharmacovigilance-oriented analytic tools (Li et al., 2026). These models help stratify reporting patterns and enrich signal-relevant contexts within spontaneous reporting data, but they do not constitute patient-level clinical prediction models and should not be interpreted as establishing biologic susceptibility or causal risk factors. A related limitation applies specifically to the interpretation of concomitant medication use in the CRS regression model. In FAERS, co-medications recorded in a report cannot always be reliably distinguished as antecedent exposures *versus* drugs administered after adverse-event onset. This is particularly important for CRS, because rescue agents such as dexamethasone and tocilizumab are commonly given after CRS develops and were among the most frequently reported co-medications in our CRS cases. Accordingly, the observed association between concomitant medication use and CRS is highly susceptible to indication bias and reverse causality, and should not be interpreted as evidence that these medications increased the pre-onset risk of CRS. Fifth, FAERS lacks sufficiently granular information on dose, step-up dosing intensity, cumulative exposure, treatment modification, and adherence (Hu et al., 2025; Kong et al., 2025). Sixth, the WHO-VigiAccess comparison should not be regarded as independent external validation, because VigiBase, the underlying source for WHO-VigiAccess, may include reports originating from FAERS and other overlapping national pharmacovigilance systems (Li J. et al., 2025). Accordingly, this component of the study is best interpreted as descriptive cross-database corroboration rather than formal validation. Finally, several less-recognized signals were supported by only a small number of reports and should therefore be regarded as hypothesis-generating observations requiring confirmation in prospective cohorts, registries, or other clinically detailed real-world datasets.

## 5 Conclusion

In this pharmacovigilance study based on FAERS, with cross-database descriptive corroboration from WHO-VigiAccess, tarlatamab showed a post-marketing reporting pattern dominated by early-onset immune-mediated and neurologic toxicities. CRS and ICANS were the most robust and clinically relevant signals, while pyrexia, dysgeusia, hypotension, and ageusia were also prominent. Several less-recognized serious events, including TLS, intestinal perforation, dyspnoea at rest, and incontinence, also warrant continued surveillance, although such sparse signals should be regarded as hypothesis-generating. Most reported events occurred early after treatment initiation, highlighting the importance of close monitoring and prompt supportive management during initial treatment cycles. In addition, machine-learning and SHAP-based analyses provided an exploratory analytic layer for stratification reporting pattern and improving model interpretability within the pharmacovigilance setting, particularly for immune-related adverse events and CRS, but they should not be interpreted as patient-level clinical prediction tools. Overall, our findings provide hypothesis-generating post-marketing safety insights and support prospective, clinically detailed real-world studies to better define the incidence, determinants, and management implications of tarlatamab-related toxicities.

## Data Availability

The original contributions presented in the study are included in the article/Supplementary Material, further inquiries can be directed to the corresponding author.

## Supplementary material

SUPPLEMENTARY FIGURE S1 Multivariable logistic regression analysis identifying high-risk factors for cytokine release syndrome-related fatal reports during tarlatamab use. **(A)** Receiver operating characteristic (ROC) curve evaluating the discriminative performance of the multivariable model. **(B)** Nomogram constructed to estimate the risk of cytokine release syndrome-related fatal reports during tarlatamab use. **(C)** Calibration curve assessing agreement between predicted probabilities and observed outcomes. TPR, true positive rate; FPR, false positive rate; MD, physician; PH, pharmacist; HP, health professional; CN, consumer; US, United States; KR, Korean; CA, Canada; JP, Japan.

SUPPLEMENTARY FIGURE S2 The ten most frequently reported concomitant drugs in CRS cases.

SUPPLEMENTARY TABLE S1 Hyperparameter tuning ranges and optimal values for 13 ML algorithms.

SUPPLEMENTARY TABLE S2 Disproportionality signal strength of adverse drug events related to tarlatamab across system organ classes in the FAERS database.

SUPPLEMENTARY TABLE S3 Metrics of 13 machine learning models in predicting tarlatamab-related immune adverse events in training (A) and test (B) sets. PPV, positive predictive value; NPV, negative predictive value.

SUPPLEMENTARY TABLE S4 Risk factors associated with tarlatamab-related cytokine release syndrome: results from univariate and multivariate logistic regression analyses in the FAERS database. MD, physician; PH, pharmacist; HP, health professional; CN, consumer; US, United States; KR, Korean; CA, Canada; JP, Japan.

SUPPLEMENTARY TABLE S5 Univariate and multivariate logistic regression analyses of risk factors associated with mortality in tarlatamab-related cytokine release syndrome cases reported in the FAERS database. MD, physician; PH, pharmacist; HP, health professional; CN, consumer; US, United States; KR, Korean; CA, Canada; JP, Japan; IL, Italy; BR, Brazil.
