# Supplementary figures and images for: Post-marketing safety of tarlatamab in small cell lung cancer based on FAERS and WHO-VigiAccess with SHAP-based interpretable machine learning analysis of immune-related adverse events
Source: Front Pharmacol. 2026 Jun 10;17:1844248. doi: 10.3389/fphar.2026.1844248 (PMC13291143; doi:10.3389/fphar.2026.1844248)

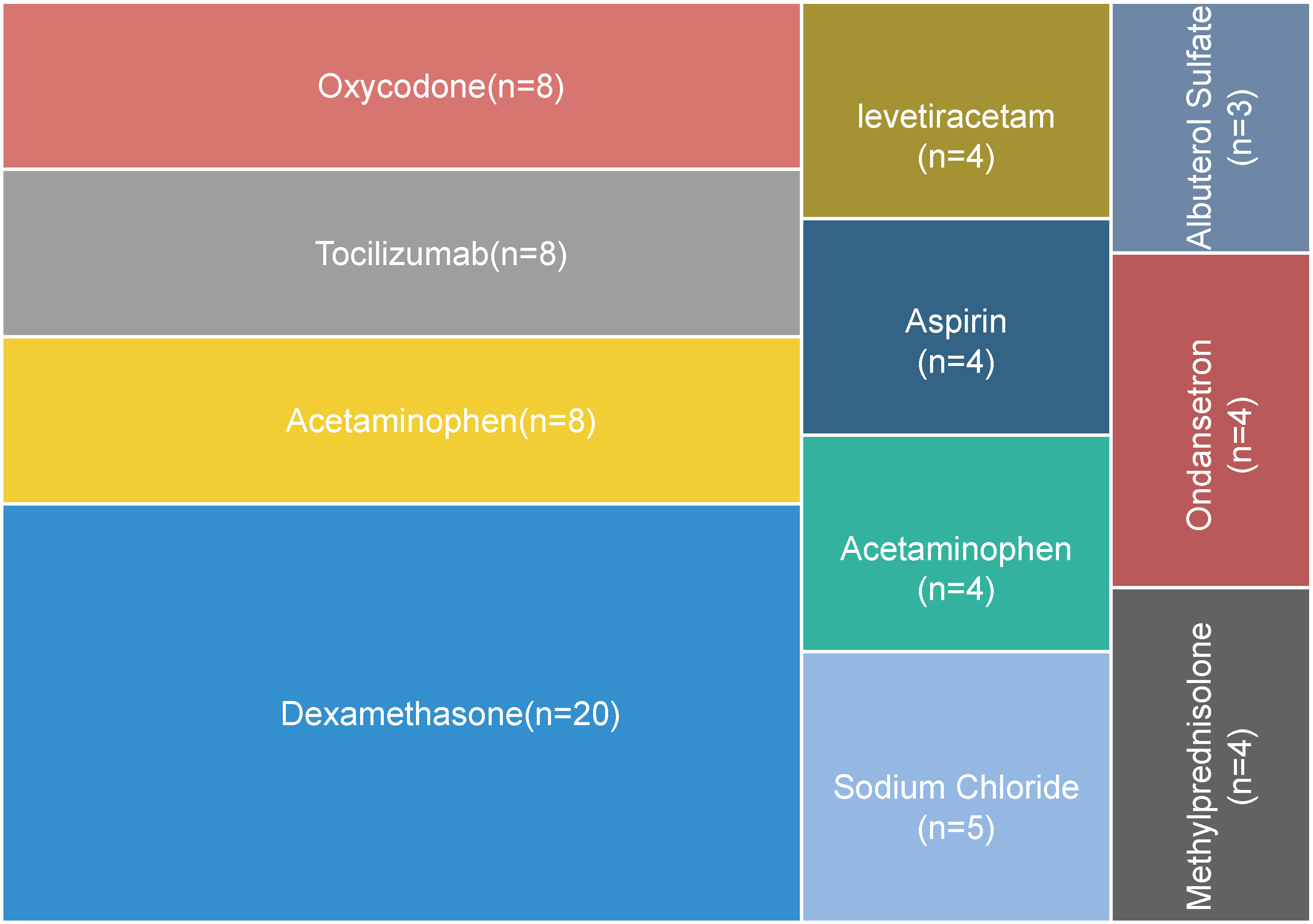

Supplement: Supplementary file 2 [file Image2.tif]

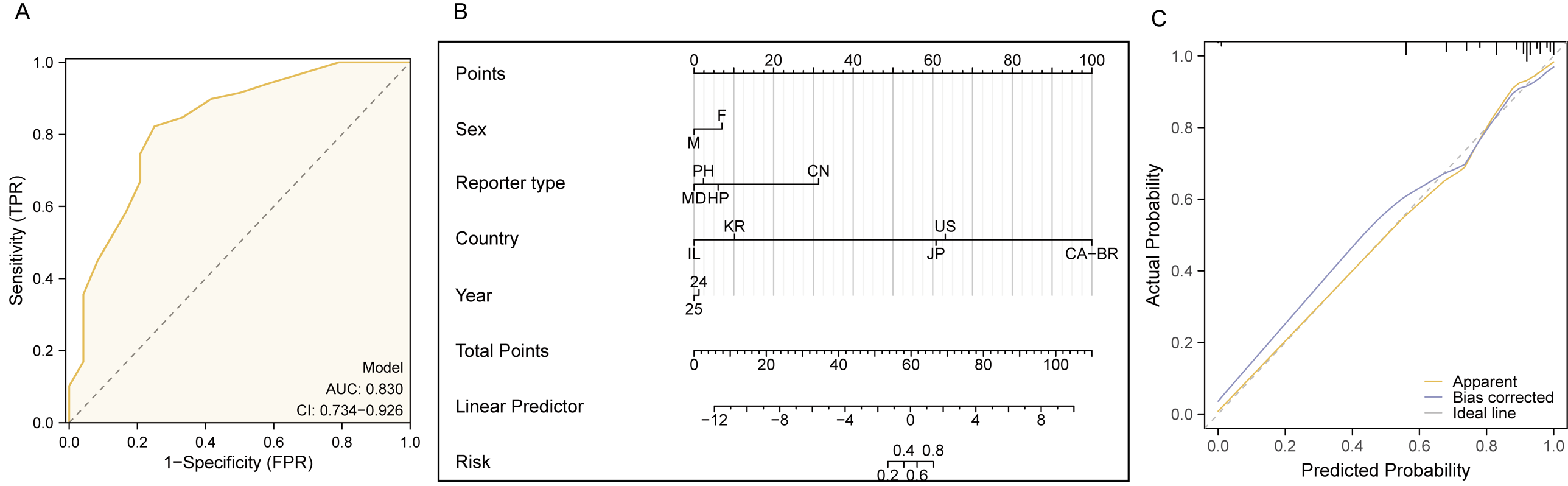

Supplement: Supplementary file 3 [file Image1.tif]

A
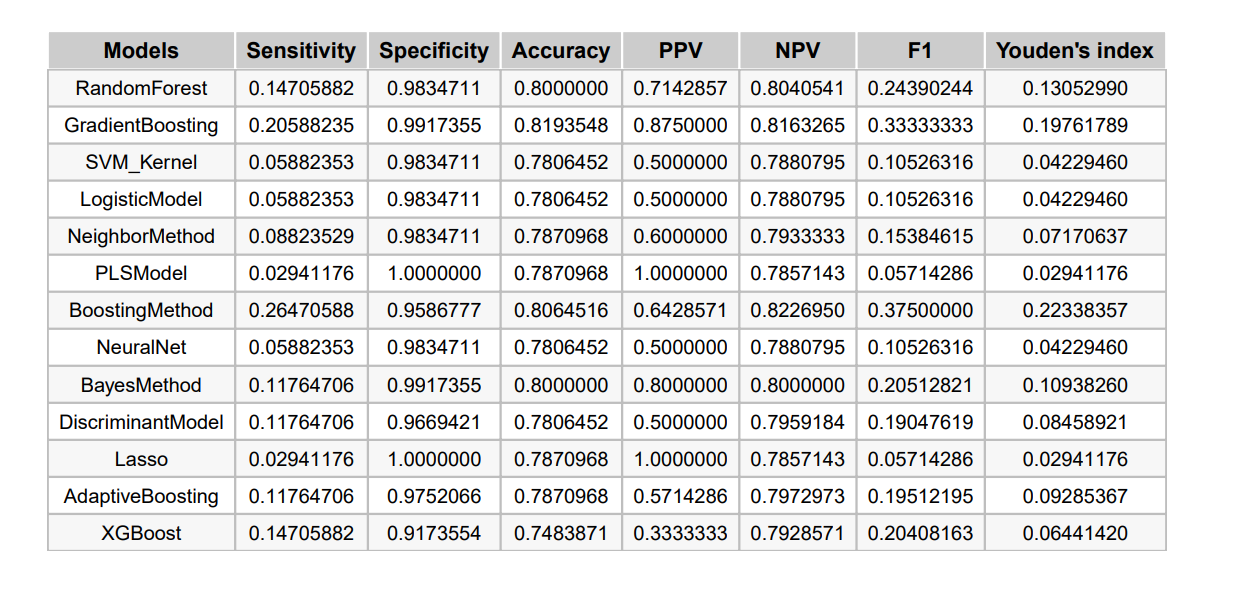


B


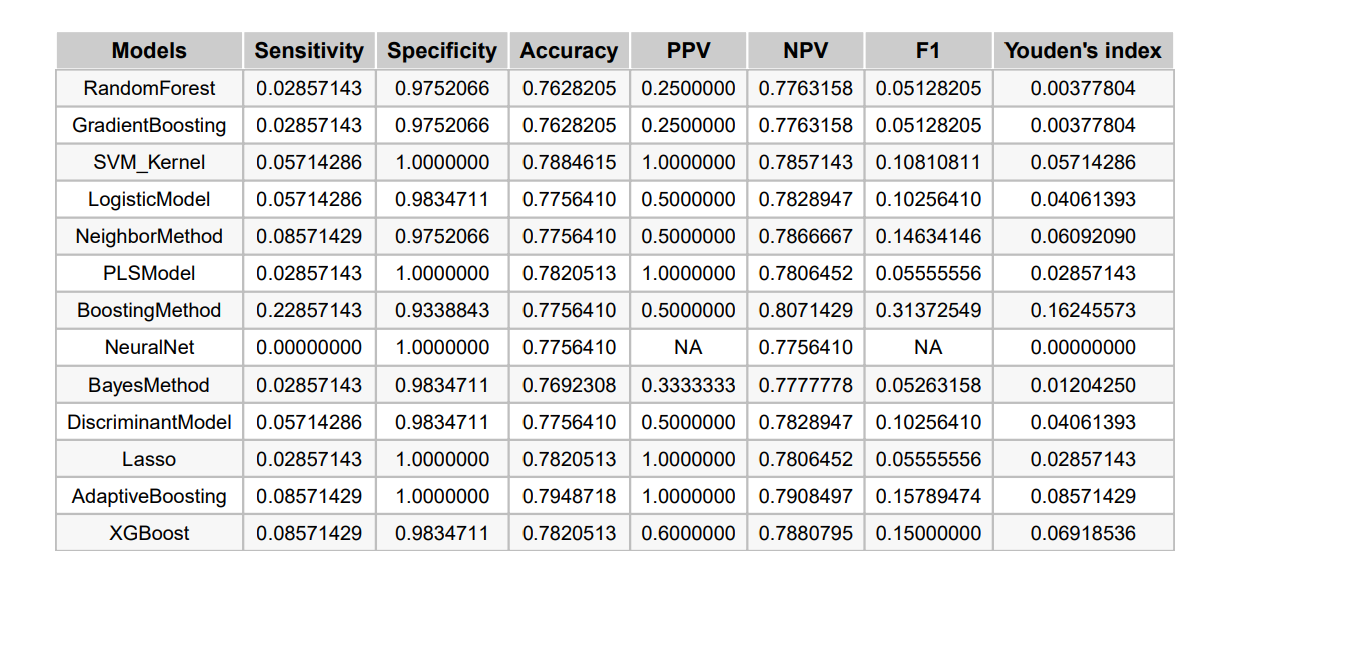

Supplement: Supplementary file 5 [file Table3.docx]
