# Supplementary material for: Post-marketing safety of tarlatamab in small cell lung cancer based on FAERS and WHO-VigiAccess with SHAP-based interpretable machine learning analysis of immune-related adverse events
Source: Front Pharmacol. 2026 Jun 10;17:1844248. doi: 10.3389/fphar.2026.1844248 (PMC13291143; doi:10.3389/fphar.2026.1844248)
Supplement: Supplementary file 4 [file Table5.docx]

| Characteristics | Total(N) | Univariate analysis | |  | Multivariate analysis | |
| --- | --- | --- | --- | --- | --- | --- |
|  |  | Odds Ratio (95% CI) | P value |  | Odds Ratio (95% CI) | P value |
| Age | 69 | 0.992 (0.971 – 1.014) | 0.482 |  |  |  |
| Sex | 142 |  |  |  |  |  |
| Male | 79 | Reference |  |  | Reference |  |
| Female | 63 | 3.673 (1.287 – 10.488) | **0.015** |  | 4.741 (1.143 – 19.664) | **0.032** |
| Reporter type | 201 |  |  |  |  |  |
| PH | 55 | Reference |  |  | Reference |  |
| HP | 60 | 1.400 (0.356 – 5.504) | 0.630 |  | 2.270 (0.477 – 10.804) | 0.303 |
| MD | 73 | 0.284 (0.099 – 0.818) | **0.020** |  | 0.592 (0.170 – 2.062) | 0.410 |
| CN | 13 | 4254481.2357 (0.000 – Inf) | 0.989 |  | 9793218.8912 (0.000 – Inf) | 0.994 |
| Country | 201 |  |  |  |  |  |
| US | 174 | Reference |  |  | Reference |  |
| KR | 4 | 0.000 (0.000 – Inf) | 0.992 |  | 0.000 (0.000 – Inf) | 0.995 |
| IL | 1 | 0.000 (0.000 – Inf) | 0.996 |  | 0.000 (0.000 – Inf) | 0.997 |
| CA | 2 | 4308335.4265 (0.000 – Inf) | 0.996 |  | 55720701.1915 (0.000 – Inf) | 0.997 |
| BR | 1 | 4308335.4251 (0.000 – Inf) | 0.997 |  | 55720701.3642 (0.000 – Inf) | 0.998 |
| JP | 19 | 0.174 (0.060 – 0.503) | **0.001** |  | 0.597 (0.134 – 2.661) | 0.499 |
| Year | 201 |  |  |  |  |  |
| 2024 | 120 | Reference |  |  | Reference |  |
| 2025 | 81 | 0.451 (0.201 – 1.014) | 0.054 |  | 0.758 (0.211 – 2.723) | 0.672 |
| Therapy | 201 |  |  |  |  |  |
| Mono | 110 | Reference |  |  |  |  |
| Combined | 91 | 0.679 (0.305 – 1.513) | 0.344 |  |  |  |
