# Supplementary material for: Post-marketing safety of tarlatamab in small cell lung cancer based on FAERS and WHO-VigiAccess with SHAP-based interpretable machine learning analysis of immune-related adverse events
Source: Front Pharmacol. 2026 Jun 10;17:1844248. doi: 10.3389/fphar.2026.1844248 (PMC13291143; doi:10.3389/fphar.2026.1844248)
Supplement: Supplementary file 6 [file Table4.docx]

| Characteristics | Total(N) | Univariate analysis | |  | Multivariate analysis | |
| --- | --- | --- | --- | --- | --- | --- |
|  |  | Odds Ratio (95% CI) | P value |  | Odds Ratio (95% CI) | P value |
| Age | 323 | 0.972 (0.952 – 0.993) | **0.010** |  | 0.981 (0.957 – 1.005) | 0.118 |
| Sex | 609 |  |  |  |  |  |
| Male | 344 | Reference |  |  |  |  |
| Female | 265 | 1.046 (0.717 – 1.527) | 0.815 |  |  |  |
| Reporter type | 941 |  |  |  |  |  |
| MD | 415 | Reference |  |  | Reference |  |
| PH | 165 | 2.342 (1.554 – 3.532) | **< 0.001** |  | 1.366 (0.638 – 2.922) | 0.422 |
| HP | 264 | 1.378 (0.939 – 2.021) | 0.101 |  | 0.717 (0.327 – 1.571) | 0.406 |
| CN | 97 | 0.725 (0.384 – 1.370) | 0.322 |  | 0.310 (0.060 – 1.606) | 0.163 |
| Country | 942 |  |  |  |  |  |
| US | 672 | Reference |  |  | Reference |  |
| Others | 43 | 0.140 (0.033 – 0.583) | **0.007** |  | 0.252 (0.053 – 1.193) | 0.082 |
| KR | 22 | 0.636 (0.212 – 1.905) | 0.419 |  | 0.385 (0.111 – 1.339) | 0.133 |
| CA | 24 | 0.260 (0.061 – 1.118) | 0.070 |  | 1.126 (0.206 – 6.159) | 0.891 |
| JP | 181 | 0.336 (0.202 – 0.557) | **< 0.001** |  | 0.362 (0.169 – 0.776) | **0.009** |
| Therapy | 942 |  |  |  |  |  |
| Mono | 601 | Reference |  |  | Reference |  |
| Combined | 341 | 1.625 (1.184 – 2.230) | **0.003** |  | 2.551 (1.353 – 4.811) | **0.004** |
| Year | 942 |  |  |  |  |  |
| 2024 | 307 | Reference |  |  | Reference |  |
| 2025 | 635 | 0.228 (0.164 – 0.316) | **< 0.001** |  | 0.450 (0.219 – 0.924) | **0.030** |
